# Terminally Truncated Isopenicillin N Synthase Generates a Dithioester Product: Evidence for a Thioaldehyde Intermediate during Catalysis and a New Mode of Reaction for Non‐Heme Iron Oxidases

**DOI:** 10.1002/chem.201701592

**Published:** 2017-08-21

**Authors:** Luke A. McNeill, Toby J. N. Brown, Malkit Sami, Ian J. Clifton, Nicolai I. Burzlaff, Timothy D. W. Claridge, Robert M. Adlington, Jack E. Baldwin, Peter J. Rutledge, Christopher J. Schofield

**Affiliations:** ^1^ Oxford Centre for Molecular Sciences and the Department of Chemistry Chemistry Research Laboratory Mansfield Road Oxford OX1 3TA UK; ^2^ Department of Chemistry and Pharmacy University of Erlangen-Nuremberg Egerlandstraße 1 91058 Erlangen Germany; ^3^ School of Chemistry The University of Sydney, NSW 2006 Australia; ^4^ Present Address: Oxford Nanopore Technologies, Oxford Science Park OX4 4GA UK; ^5^ Present Address: The Brattle Group Level 15 5 Martin Place Sydney, NSW 2000 Australia; ^6^ Present Address: Immunocore Limited 101 Park Drive, Milton Park Abingdon OX14 4RY UK

**Keywords:** biosynthesis, enzymes, metalloenzymes, non-heme oxygenase, oxidoreductases, penicillin

## Abstract

Isopenicillin N synthase (IPNS) catalyses the four‐electron oxidation of a tripeptide, l‐δ‐(α‐aminoadipoyl)‐l‐cysteinyl‐d‐valine (ACV), to give isopenicillin N (IPN), the first‐formed β‐lactam in penicillin and cephalosporin biosynthesis. IPNS catalysis is dependent upon an iron(II) cofactor and oxygen as a co‐substrate. In the absence of substrate, the carbonyl oxygen of the side‐chain amide of the penultimate residue, Gln330, co‐ordinates to the active‐site metal iron. Substrate binding ablates the interaction between Gln330 and the metal, triggering rearrangement of seven C‐terminal residues, which move to take up a conformation that extends the final α‐helix and encloses ACV in the active site. Mutagenesis studies are reported, which probe the role of the C‐terminal and other aspects of the substrate binding pocket in IPNS. The hydrophobic nature of amino acid side‐chains around the ACV binding pocket is important in catalysis. Deletion of seven C‐terminal residues exposes the active site and leads to formation of a new type of thiol oxidation product. The isolated product is shown by LC‐MS and NMR analyses to be the ene‐thiol tautomer of a dithioester, made up from two molecules of ACV linked between the thiol sulfur of one tripeptide and the oxidised cysteinyl β‐carbon of the other. A mechanism for its formation is proposed, supported by an X‐ray crystal structure, which shows the substrate ACV bound at the active site, its cysteinyl β‐carbon exposed to attack by a second molecule of substrate, adjacent. Formation of this product constitutes a new mode of reaction for IPNS and non‐heme iron oxidases in general.

## Introduction

**Scheme 1:**
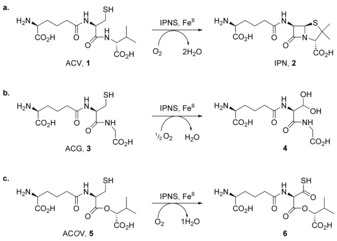
IPNS‐catalysed oxidation of: a) its natural substrate ACV **1** to the bicyclic penam IPN **2** by means of a four electron (4e) oxidation in which one molecule of O_2_ is consumed per molecule of ACV, which loses four hydrogen atoms; b) the analogue ACG **3** to the shunt metabolite **4** (a hydrated aldehyde) by means of a two electron (2e) oxidation;^13^, ^19^ and c) the depsipeptide analogue AC*O*V **5** by means of another 4e process, in which all the oxidizing potential of O_2_ is directed at the cysteinyl β‐carbon to form thiocarboxylic acid **6** (as characterised crystallographically).^15^

Isopenicillin N synthase (IPNS) catalyses the 4‐electron oxidation of the tripeptide l‐δ‐(α‐aminoadipoyl)‐l‐cysteinyl‐d‐valine (ACV, **1**) to give bicyclic isopenicillin N (IPN, **2**) with concomitant reduction of one molecule of dioxygen to two water molecules (Scheme 1 a).^1^

IPNS is part of the superfamily of iron(II) dependent oxidases.^2^, ^3^, ^4^ Crystal structures of the *Aspergillus nidulans* protein^5^, ^6^, ^7^ show IPNS to contain the double‐stranded β‐helix fold (DSBH, also known as a “jelly‐roll”, cupin or jumonji C (JmjC) fold) characteristic of the 2‐oxoglutarate‐dependent oxygenase superfamily (Figure S1, Supporting Information).^8^ These structures reveal the presence of the characteristic “2‐His‐1‐carboxylate” facial triad of metal‐ligating residues (His214, Asp216 and His270), an arrangement present in many non‐heme iron(II) dependent oxidases.^9^, ^10^, ^11^ The iron(II) centre has an octahedral coordination geometry, with the other potential binding sites occupied at various points of the catalytic cycle by the amide side‐chain of Gln330, one or two water molecules, the sulfur of the substrate **1** thiol, the isopropyl group of the substrate **1** d‐valine (sitting within van der Waals contact of iron), molecular oxygen and reactive species derived from oxygen.^6^, ^7^ ACV **1** is bound through thiolate ligation to iron and by electrostatic interactions, including a salt bridge from the aminoadipoyl carboxylate to Arg87, hydrogen bonding between the valinyl carboxylate, Tyr189 and Ser281, and hydrophobic interactions involving the isopropyl group of d‐valine and a pocket formed by the side‐chains of Pro283, Leu223, Leu231, Val272 and Thr221.^6^, ^7^

The mechanism of IPNS has been studied using a wide range of ACV analogues in solution,^12^ in crystallisation studies,^5^, ^6^, ^13^, ^14^ and by turnover within the crystalline protein (with reaction initiated by exposing anaerobic crystals to pressurised oxygen gas).^7^, ^15^, ^16^, ^17^, ^18^ IPNS catalyses an array of different oxidation reactions both in solution and in crystals; many of the ACV analogues studied, particularly those altered in the third residue (valine), are oxidised to alternative cyclic and acyclic products. For example, turnover of l‐δ‐(α‐aminoadipoyl)‐l‐cysteinyl‐glycine (ACG **3**, glycine replaces valine) does not generate a bicyclic product; the 5‐membered thiazolidine ring cannot form, so the monocyclic β‐lactam intermediate opens, giving a 2‐electron oxidised shunt product in which the cysteine residue is oxidised to an aldehyde (observed as the hydrate **4**) through a two‐electron oxidation (Scheme 1 b).^13^, ^19^ By contrast, the depsipeptide substrate analogue δ‐(l‐α‐aminoadipoyl)‐l‐cysteine d‐α‐hydroxyisovaleryl ester (AC*O*V **5**, ester replaces amide between cysteine and valine), cannot form a β‐lactam intermediate.^15^ Instead, both oxidising equivalents are directed at the cysteinyl β‐carbon, which is converted from the thiol in **5** to the thiocarboxylic acid **6** through 4‐electron oxidation (Scheme 1 c).

**Figure 1:**
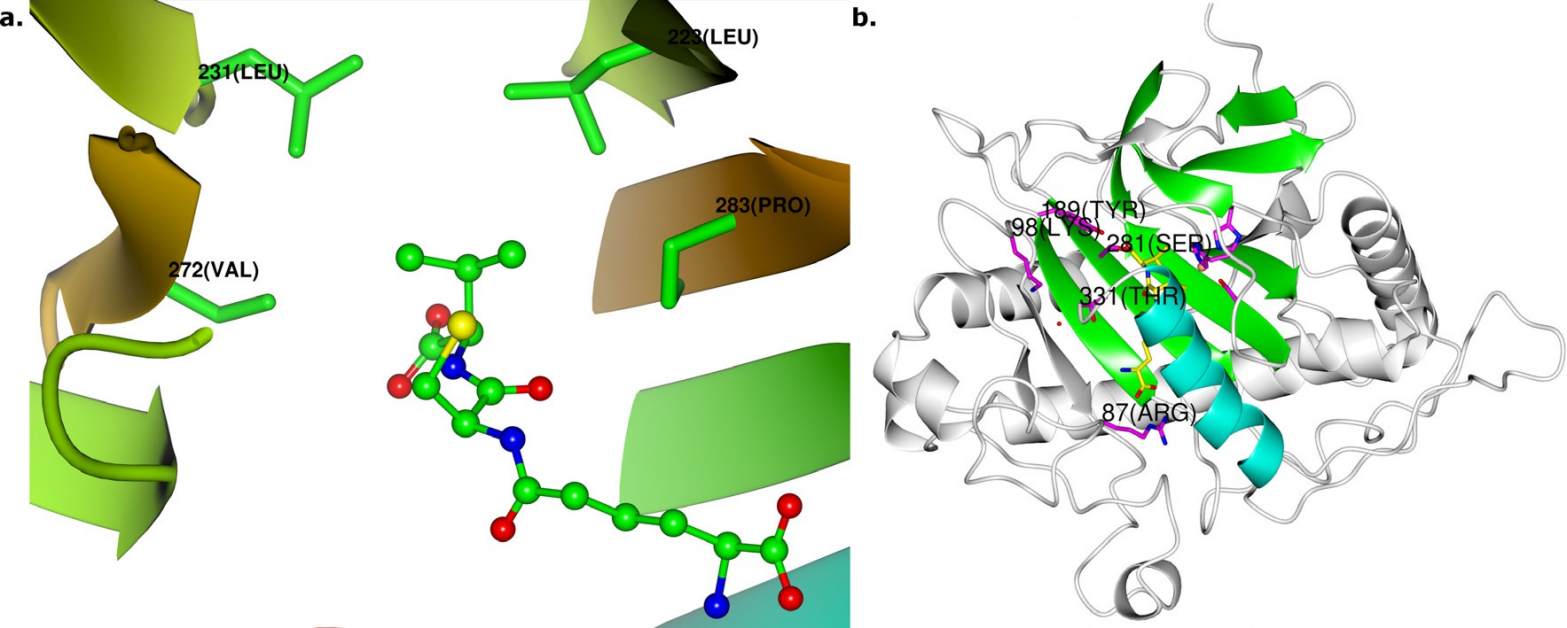
a) Key residues in the hydrophobic binding pocket around the valinyl side chain of ACV **1** at the active site of the IPNS:Fe^II^:ACV complex (PDB ID 1BK0).^6^ The side‐chains of Leu223 (at “1 o′clock” relative to ACV valine), Leu231 (11 o′clock), Val272 (9 o′clock) and Pro283 (3 o′clock) are shown. b) Structure of the whole IPNS:Fe^II^:ACV complex, highlighting the core double‐stranded β‐helix fold (green), and the C‐terminal α‐helical region, residues 313–331 (cyan). Note the position of the six C‐terminal residues Asn326‐Thr331 at the top of this helix, enclosing ACV in the active site. Iron is in orange, key substrate‐binding residues are magenta, and ACV **1** is yellow. See also Figure S1 in the Supporting Information for comparison of this structure with the structure of IPNS without substrate bound.

These and other studies, including a range of computational and spectroscopic investigations,^20^, ^21^, ^22^, ^23^, ^24^ have led to a detailed chemical understanding of IPNS catalysis, and of the roles played by some key active site residues beyond the iron‐binding residues.^25^ The side‐chain amide carbonyl oxygen of Gln330, the penultimate residue of the enzyme, ligates to the active site metal only in the absence of substrate, as observed in the structure of the IPNS:Mn^II^ complex (Figure S1 a).^5^ In the structure of the anaerobic IPNS:Fe^II^:ACV complex^6^ and structures with substrate analogues,^13^, ^26^, ^27^, ^28^, ^29^ the side‐chain of Gln330 is displaced from the metal to enable ligation of the substrate sulfur. Movement of Gln330 enables formation of a salt bridge between the carboxylate of Thr331 and Lys98, with the six C‐terminal residues (Asn326‐Thr331) taking up a position that extends the final helix (α‐10), thus enclosing the substrate in the active site (Figure 1 b and Figure S1). Gln330 is highly conserved across IPNS proteins from different species, but it is not essential for catalysis.^30^ Changing Gln330 to Ala or Leu, or deleting a few amino acids (two or six) from the C‐terminal, leads to significant reductions in specific activity (*k*_cat_ ca. 10 % of wildtype), but the enzyme remains catalytically viable; deleting eight residues ablates activity.^30^ Reports concerning 2‐oxoglutarate oxygenases, which are from the same structural subfamily as IPNS, have observed that their C‐terminal region can exert important control over reactivity. For instance, the C‐terminal residues of gibberellin 20‐oxidases are involved in regulation of specificity,^31^ deleting the last five residues of deacetoxycephalosporin synthase (DAOCS) yields an enzyme that converts the 2‐oxoglutarate co‐substrate to succinate at the same rate as the wildtype but which does not couple this to oxidation of the primary substrate (penicillin N),^32^ and the C‐terminal region is important in catalysis by the hypoxia‐sensing enzyme prolyl hydroxylase domain 2 (PHD2).^33^

To better characterise the hydrophobic binding pocket around the ACV valine residue (Figure 1 a) and to investigate the role of the dynamic C‐terminus in protecting the IPNS active site after substrate binding, we constructed a range of IPNS variants, determined their catalytic competencies, and investigated the products formed when these modified proteins react with ACV. The interaction between substrate ACV and binding pocket was investigated by changing Leu223, Leu231, Val272 and Pro283, whereas interactions between the protein C‐terminal and the substrate were investigated by substituting a residue that contributes indirectly to substrate binding (Lys98), and by extending (*332Q) or shortening (I325*) the terminal residues. The choice and design of mutants is discussed further in the Supporting Information. Unexpectedly, deletion at the C‐terminus enables formation of an entirely new type of enzyme‐catalysed thiol oxidation product. We report the characterisation of this enzymatically unprecedented product as the ene‐thiol tautomer of a dithioester, made up from two molecules of ACV linked between the thiol sulfur of one tripeptide and the oxidised cysteinyl β‐carbon of the other.

## Results and Discussion

### Investigation of hydrophobic substrate binding pocket

**Figure 2:**
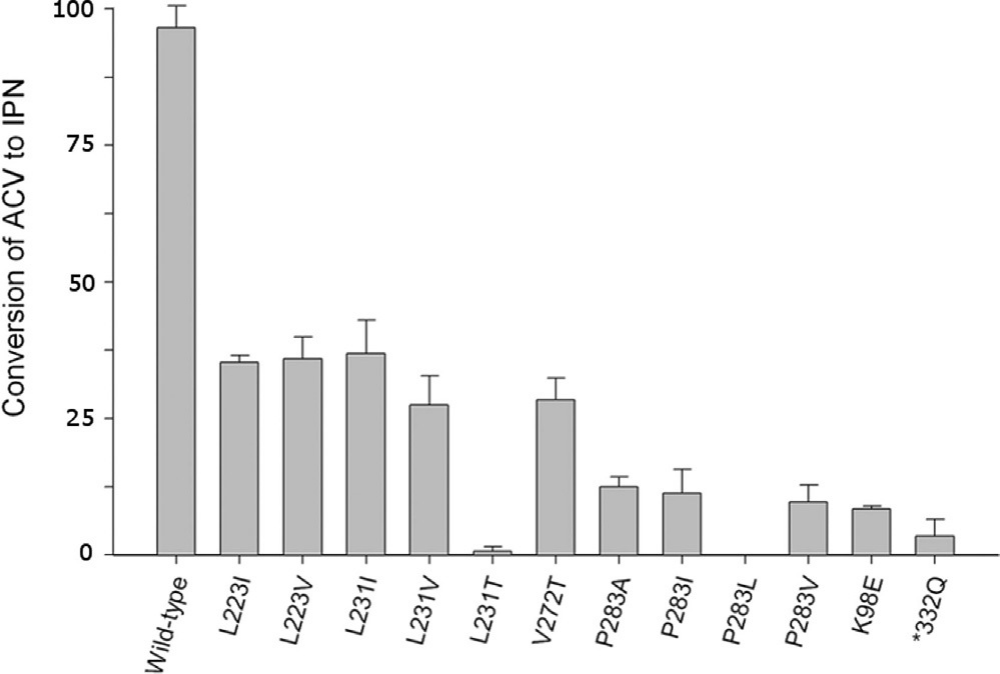
Activity of mutant IPNS proteins as percentage of wildtype activity, measured by HPLC analysis of the extent of IPN **2** production from ACV **1** after 10 min turnover reaction. Figure shows total turnovers of ACV to IPN in 10 min at 28 °C (for further details of assay conditions, see the Experimental Section).

IPNS variants were constructed as shown in Tables S1 and S2 (see the Supporting Information) and assayed for activity in terms of IPN production from ACV. The results reveal that alteration of the hydrophobic binding pocket (Figure 1) reduces the capacity of the enzyme to function efficiently (Figure 2), with some apparent variations for specific amino acids. For a more detailed discussion of these substitutions and their effects, see the Supporting Information.

### Product distributions with the “tailless” I325* mutant

Previous work with I325* IPNS has shown it retains the ability to convert ACV **1** to IPN **2**, but with only 10 % of wildtype activity.^30^ Further examination of elution profiles from HPLC analyses of ACV turnover by the I325* truncated variant revealed a new “split” peak, due to a new product designated henceforth as compound **7**. This new product eluted from the C18 column between IPN **2** and ACV thiol **1** (Figure S2); the production of **7** increased with time at approximately the same rate as IPN **2**, and was not observed in control reactions using heat inactivated enzyme. This new product was generated in sufficient quantities to allow isolation and characterisation. The split peak observed was not resolvable under all HPLC conditions screened and was collected as a single fraction. Subsequent analysis showed the fraction to contain an equilibrating mixture of thio‐keto/enol tautomers (see below). [ACV]_2_ (disulfide) was observed eluting later than ACV thiol **1**, IPN **2** and the new compound **7**, whereas several other new species were also observed but were not produced in sufficient quantities to enable characterisation.

Larger quantities of **7** were obtained by repeating the incubation and employing multiple chromatographic isolations, generating sufficient quantities of pooled **7** for MS and NMR analyses. A second HPLC purification step (using a higher concentration of ammonium bicarbonate, see the Experimental Section) was required to separate **7** from traces of co‐eluting ACV thiol **1**. Typically ACV **1** (100 mg) yielded only very small quantities of **7** (<1 mg) due to the relatively poor turnover of ACV by I325* IPNS. Some product loss was also incurred during isolation, by virtue of repeated HPLC purification steps and the apparent instability of **7**. During these investigations, it was noted that **7** displayed a characteristic absorption band at *λ*_max_=317 nm (Figure S3 a, Supporting Information), as confirmed during subsequent analyses (Supporting Information).

High resolution mass spectrometric analysis (positive ion mode) gave a molecular ion for **7** at *m*/*z* 723.2697 (i.e., [MH]^+^ for a neutral molecule of molecular formula C_28_H_46_N_6_O_12_S_2_), suggesting **7** is an oxidised dimer of two ACV molecules. (ACV **1** has the molecular formula C_14_H_25_N_3_O_6_S and [MH]^+^ with *m*/*z* 364.1542; the ACV disulfide [ACV]_2_ has formula C_28_H_48_N_6_O_12_S_2_; i.e., [MH]^+^ with *m*/*z* 725.2850.) The mass of **7** did not change after reaction with excess sodium borohydride, indicating that the new compound does not contain a disulfide S−S. Quantitative reaction of **7** with Ellman's reagent (5,5′‐dithiobis‐(2‐nitrobenzoic acid, DTNB),^34^ using *ϵ*=13 600 m ^−1^ cm^−1^ for the TNB^−^ anion and quantifying against a dithiothreitol standard, indicated the presence of a single thiol group in compound **7**.

The reaction of **7** with dithiothreitol (DTT) led to the isolation of ACV thiol **1** (assigned by comparison of HPLC retention times and mass spectra with an authentic sample) and a species of *m*/*z* 514, consistent with a species ([MH]^+^) arising from DTT and the desaturated half of **7** (i.e., ACV‐2 H, Figure S4). The absorption band at 317 nm in the original isolated species is maintained on reaction with DTT. Iodoacetamide reacts only once with **7** to give a product of mass 780 Da ([MH]^+^), i.e. consistent with the addition of one equivalent of CH_2_CONH_2_ in a single alkylation reaction, shifting the absorbance maximum to 297 nm. When both iodoacetamide and DTT are simultaneously added to **7**, all species described in this paragraph are observed (Figures S4 and S5 and Table S3 in the Supporting Information).

**Figure 3:**
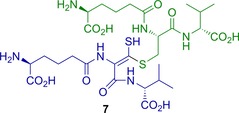
Proposed structure for **7** as assigned by NMR and MS analyses. (*S*)‐2‐Amino‐6‐(((*S*)‐3‐(((*E*)‐2‐((*S*)‐5‐amino‐5‐carboxypentanamido)‐3‐(((*R*)‐1‐carboxy‐2‐methylpropyl)amino)‐1‐mercapto‐3‐oxoprop‐1‐en‐1‐yl)thio)‐1‐(((*R*)‐1‐carboxy‐2‐methylpropyl)amino)‐1‐oxopropan‐2‐yl)amino)‐6‐oxohexanoic acid **7** is an oxidised dimer of two molecules of ACV **1**: one ACV (green) is intact (but for its thiol S−H), the other (blue) has been doubly oxidised at cysteine (from thiol to thiocarboxylic acid oxidation state) and contains an α,β‐desaturated cysteinyl residue. Note that the *E*/*Z* stereochemistry of the alkene in **7** (and analogous products) is not defined by the NMR analyses and may equilibrate through tautomerism. Stereochemistry at all other positions is assumed to be as in ACV **1**.

These data, combined with further analysis by NMR (see below), suggested that **7** is an oxidised ACV dimer, in which one ACV has been doubly oxidised at cysteine (i.e., undergone a four‐electron oxidation) and contains an α,β‐desaturated cysteinyl residue (i.e., is predominantly the ene‐thiol tautomer of a dithioester) and the second ACV is covalently linked through its thiol‐derived sulfur to the cysteinyl β‐carbon of the first ACV (Figure 3 **)**.

**Figure 4:**
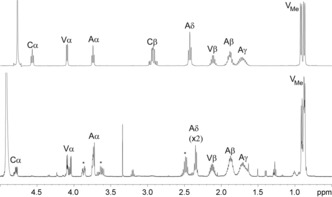
^1^H NMR spectra of ACV starting material (upper spectrum, 400 MHz), and the oxidised product **7** (lower spectrum, 500 MHz). Assignments are for α‐, β‐, γ‐, δ‐ and methyl protons on the aminoadipoyl (A), cysteinyl (C) and valinyl (V) portions of the substrate. The asterisks indicate new peaks in the spectrum of **7** compared to ACV **1**.

Analysis of the 500 MHz ^1^H NMR spectrum of **7** (Figure 4, Table S4 in the Supporting Information), along with COSY and HMQC spectra (Figures S5 and S6 a in the Supporting Information), enabled identification of spin‐coupled systems corresponding to two valinyl moieties and two aminoadipoyl components, but only one cysteine. The aliphatic region of the 1D ^13^C NMR spectrum (data not shown) supported this assignment; however, the limited amount of material available meant that the signal‐to‐noise ratio of this spectrum was too low to reliably reveal the carbonyl resonances or other quaternary centres. Further information was therefore sought in the HMBC long‐range heteronuclear correlation spectrum **(**Figures S6 b and S7). Though most of the correlations observed were as expected for the parent ACV **1**, two additional correlations are of particular note. The first was from the valine α‐proton of the modified half of **7** (blue in Figure 3) to the carbonyl of the modified cysteine. This carbonyl resonance is seen at 166.8 ppm, upfield relative to the “intact” ACV cysteine carbonyl at 171.4 ppm; such an upfield shift is consistent with this carbonyl being adjacent to the unsaturated ene‐thiol functionality in **7**. The second noteworthy correlation is from both β‐protons of the intact (i.e., green) cysteine to a carbon at 229.7 ppm, a resonance that is consistent with the thiocarbonyl carbon of a dithioester, i.e. the “keto” form of thio‐enol **7**.^35^, ^36^, ^37^ This correlation is indicative of a new ‐CH_2_‐S‐C‐S linkage derived from the cysteine groups of two ACV building blocks. The presence of such a linkage is consistent with the molecular formula deduced above (C_28_H_46_N_6_O_12_S_2_) and the outcome of reactions with DTT and iodoacetamide, that is: an ACV dimer containing four fewer hydrogens than two fully reduced ACV molecules, in which three hydrogens have been removed from the modified cysteinyl moiety (blue) and only one from the other cysteine (green).

**Figure 5:**
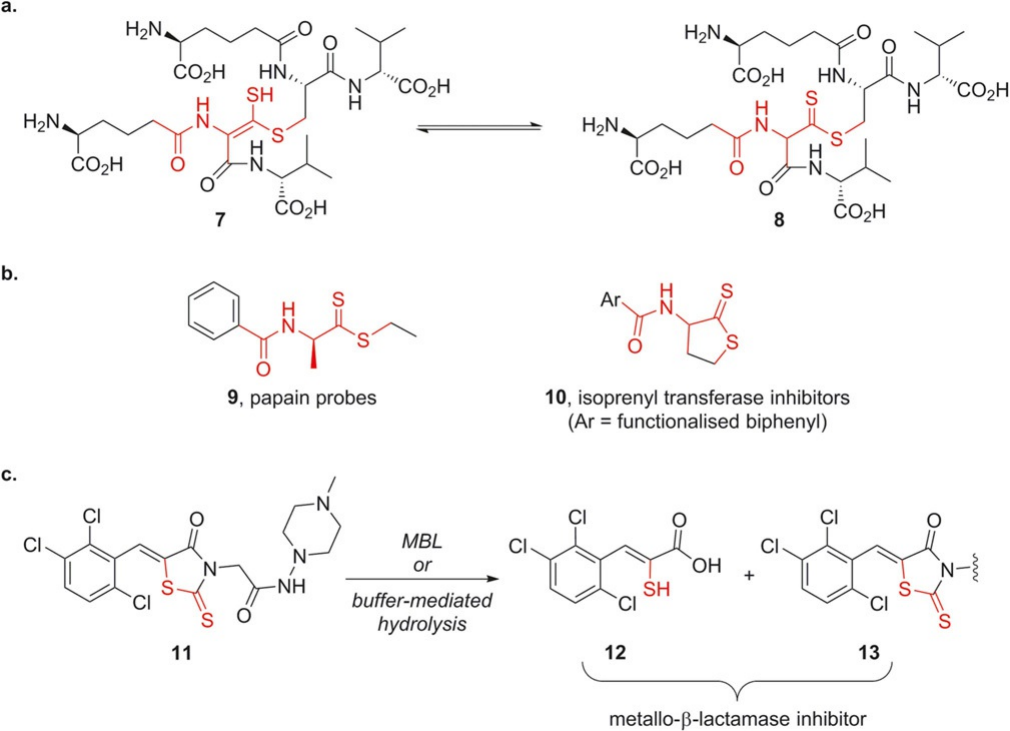
a) NMR spectroscopy experiments indicate that the ene‐thiol **7** (of undetermined alkene stereochemistry) exists in equilibrium with thiocarbonyl tautomer **8**; b) previously reported compounds **9** and **10** that include the α‐amido‐thio‐ene‐thiol motif seen in **7**, or dithioester **8**; c) rhodanine derivative ML302 **11** is a potent metallo‐β‐lactamase (MBL) inhibitor, acting via fragmentation to **12** and **13**. See the main text for discussion of results with compounds **9**–**13**.

The NMR data thus suggest that **7** exists in a thio‐keto/enol equilibrium with the thiocarbonyl form **8** (Figure 5 a), consistent with the split peak seen in the HPLC elution profile. This means that the α‐proton of the modified cysteine is exchanged for deuterium in the thio‐keto form under the solvent conditions used for NMR analysis. This observation, combined with its remoteness from non‐exchangeable hydrogens, prevents direct observation of the α‐carbon of the modified cysteine residue in the HMQC, HMBC and direct ^13^C NMR spectra.

Investigating the literature for previously reported exemplars of the core α‐amido‐dithioester or ene‐thiol tautomer seen in the new compound **7**/**8** (the region highlighted in red in Figure 5 a) returns a limited number of related structures (Figure 5 b). In a biological context, ene‐thiols are intermediates in the biosynthesis of the dithiopyrrolone core of antibiotics such as holomycin,^38^ and thioenols/thioaldehydes are intermediates in catalysis by cysteine‐derivative decarboxylases that are involved in phosphopantetheine and lantibiotic biosynthesis.^39^ Amino acid and dipeptide dithioester derivatives such as **9** have been used as probes to study mechanistic aspects of the cysteine protease papain,^40^, ^41^ whereas arylated cyclic compounds **10** have been explored as inhibitors of protein isoprenyl transferases.^42^ Interestingly, ene‐thiols have been identified as potent inhibitors of di‐Zn^II^ ion‐dependent metallo‐β‐lactamases (MBLs), through hydrolytic fragmentation of rhodanine derivative **11** (ML302) to generate the ene‐thiol **12** and compound **13** (Figure 5 c).^43^ Thus, although there is no direct evidence for the biological production of an oxidase‐catalysed ene‐thiol product, it is a conceivable mechanism for the production of metallo‐β‐lactamase inhibitors in order to potentiate β‐lactam antibiotic activity.

### Further characterisation of the I325* mutant

Further experiments incubating the I325* variant with ACV **1** and the di‐ or tripeptide analogues *N*‐acetyl‐l‐Cys‐l‐Val **14**, *N*‐acetyl‐l‐Cys‐Gly **15**, *N*‐acetyl‐l‐Ser‐l‐Val **18**, or l‐δ‐(α‐aminoadipoyl)‐l‐cysteinyl‐d‐*allo*‐isoleucine **19** (Figure S9, Table S5, Supporting Information) show that in the presence of ACV, the truncated enzyme can form ene‐thiol structures analogous to compound **7** only if the added ACV analogue contains a cysteinyl moiety. Thus, when I325* was incubated with a mixture of ACV **1** and either *N*‐acetyl‐l‐Cys‐l‐Val **14** or *N*‐acetyl‐l‐Cys‐Gly **15**, it reacted to give the analogous cysteine‐oxidised ACV/dipeptide conjugates **16** and **17** in addition to **7**, whereas with a mixture of ACV and *N*‐acetyl‐l‐Ser‐l‐Val **18** it did not (Figure S9, Supporting Information). None of these dipeptides reacted without ACV being present (this is perhaps unsurprising, as none are substrates for wildtype IPNS) and corresponding experiments with l‐cysteine itself did not give an analogous dimerised product. However, l‐δ‐(α‐aminoadipoyl)‐l‐cysteinyl‐d‐*allo*‐isoleucine **19** reacted to give a cysteine‐oxidised dimer **20**, analogous to **7** (Figure S9 d). Thus, it seems that I325* binds ACV **1** in a manner similar to the wildtype enzyme, to give an intermediate that can also react with related thiols from solution.

**Figure 6:**
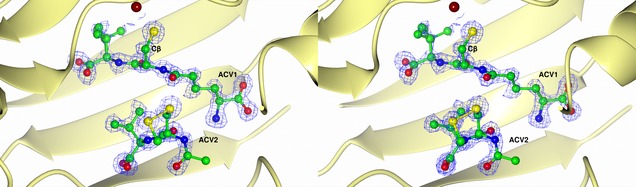
Stereoview representation showing atom positions and electron density for the substrate (ACV1) at the active site of the I325*IPNS:Fe^II^:ACV complex, with a 2mF_0_‐DF_c_ (σ_A_) electron density map shown in blue at 1.5 σ for the substrate. The catalytic metal is shown near the top of the figure (orange sphere), proximate to the thiolate sulfur and valinyl isopropyl group of ACV1. (Note that this isopropyl group likely has multiple conformations, indicating greater rotational freedom than is seen with the wildtype protein, in structure 1BK0.) Electron density due to the second ACV molecule (ACV2) occupies the position that is taken up by the tail of the intact enzyme (1BK0). Additional electron density adjacent to the thiol sulfur of ACV2 is thought to be due to partial occupancy by an ACV‐methane thiol disulfide (shown in this image overlaying the valinyl isopropyl group of ACV2), discussed in more detail in the main text. Atom colours: carbon=green; nitrogen=blue; oxygen=red; sulfur=yellow; iron=dark orange.

To investigate the mechanism of formation of the ene‐thiol products, a crystal structure for I325* in complex with iron(II) and ACV was determined (see PDB ID 2BJS, Figure 6, Figure S10 and Table S6 in the Supporting Information). The protein chain in the I325* crystal structure adopts the same fold as the wildtype IPNS:Fe^II^:ACV complex (PDB ID 1BK0), the active site iron(II) is bound by the 2‐His‐1‐carboxylate facial triad of protein‐derived ligands (the side‐chains of His214, Asp216 and His270).^9^, ^10^ The “primary” substrate molecule (ACV1) is bound in a similar manner as for the wildtype IPNS: Fe^II^:ACV complex, that is, is held by a salt bridge from its aminoadipoyl carboxylate to the side‐chain of Arg87, ligation of the cysteinyl thiolate to iron, and hydrogen bonding between the valinyl carboxylate and several protein side‐chains (as seen with the natural substrate and analogues).^6^, ^7^, ^13^, ^28^ Several minor changes are evident relative to the wildtype protein, including minor alterations in the positions of the aminoadipoyl and valinyl side chains of the active site‐bound substrate, and a small rotation in the side‐chain of Phe211 relative to the wildtype structure (Figure S10 in the Supporting Information). The position of this side‐chain has previously been observed to change upon turnover of tripeptide substrates to bicyclic penam products, and it has been proposed that—like the C‐terminal helix—this benzyl group functions as a shield to the active site.^7^, ^44^ The idea of a Phe side‐chain functioning to “gate” the active site of a metalloenzyme has also been posited to operate in bacterial persulfide dioxygenases (Fe^II^‐dependent members of the metallo‐β‐lactamase superfamily, which oxidise glutathione persulfide to sulfite and glutathione), in which the side‐chain of Phe184 has been observed to occupy alternative conformations in the presence and absence of glutathione.^45^

Notably, in the I325* structure (Figure 6) the β‐carbon of the ACV1 cysteine is clearly exposed to the external solution due to the absence of the C‐terminal helix that would otherwise cover this region. Furthermore, density consistent with a second ACV molecule (ACV2) is observed near the primary, iron‐bound ACV (ACV1). This ACV2 sits in an area of the structure close to the region that is occupied by residues Gly329 and Gln330 in structure 1BK0 of the intact enzyme; presumably in the wildtype protein, these residues prevent entry of “ACV2” to the active site, so the cysteine‐oxidised product **7** is not seen in wildtype incubations. However, it is perhaps possible that binding of “ACV2” in this site may in part reflect the workings of a substrate capture mechanism. The valinyl and cysteinyl side‐chains of ACV2 appear to be well‐ordered, but electron density for the aminoadipoyl residue was not resolved beyond its Cα atom. This observation maps nicely to the results of I325* incubations with ACV analogues **14**, **15** and **18**, which showed that the aminoadipoyl moiety can be replaced with an acetyl group, but that a cysteinyl residue linked to a second amino acid are required for formation of cysteine‐oxidised dimeric products like **7** (see above and Figure S9, Supporting Information). Extra electron density is evident adjacent to the cysteinyl sulfur of ACV2; the size and shape of this density suggest another sulfur atom (*S′*) bound in the mode of a disulfide linkage. A further very small peak of density is apparent at a position that likely corresponds to another atom (*C′*) covalently bound to the additional sulfur *S′*. We postulate that the electron density observed around ACV2 in the second ACV “binding site” of I325* IPNS corresponds to partial occupancy by ACV thiol **1**, and partial occupancy by the disulfide of ACV and methane thiol, which is an impurity present in the fermentation‐derived ACV **1**.^46^

### A mechanism for formation of the oxidised product 7

**Figure 7:**
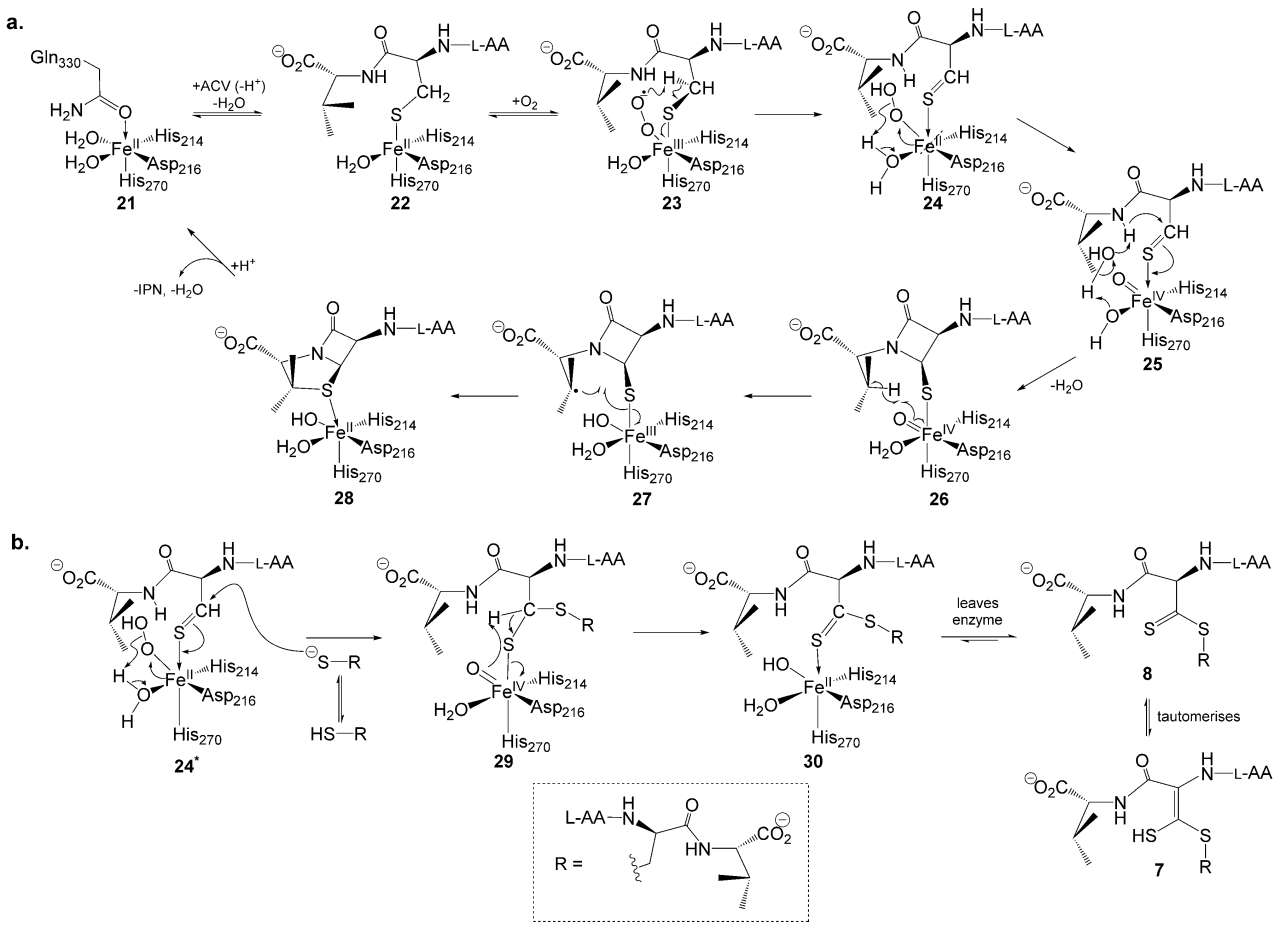
Proposed mechanisms for the reactions of wildtype and I325* IPNS with ACV **1**. a) Reaction of wildtype IPNS with ACV (adapted from ref. 25). This scheme shows the “ligand donor” model for peroxide cleavage (**24**→**26**), in which the peroxide first reacts with the adjacent water molecule at iron, and the resulting iron‐bound hydroxide then mediates deprotonation of the cysteinyl‐valine amide through a proton shuttle. Recent computational studies^20^, ^21^, ^48^ suggest this route is more likely than alternative “substrate donor” model, in which the peroxide species directly removes the N−H proton of the cysteinyl‐valine amide. b) Proposed mechanism for the conversion of ACV **1** to oxidised product **7** by the I325* mutant, in which the putative thioaldehyde intermediate 24* is intercepted by a second molecule of ACV **1**. See the main text for further details of **21**–**30**; l‐AA=l‐δ‐(α‐aminoadipoyl).

The consensus mechanism for the IPNS‐mediated transformation of ACV **1** to IPN **2** (Figure 7 a)^6^, ^7^, ^24^, ^25^ begins with displacement of Gln330 from the active site of the apo‐enzyme complex **21** as first ACV and then O_2_ bind to iron to form the complexes **22** and then **23**. Oxygen binds end‐on in the site opposite Asp216,^6^ avoiding the bridged binding mode seen with other non‐heme iron enzymes and thus enabling oxidase activity (the primary reaction pathway for IPNS) in preference to oxygenase activity. Oxygen sits adjacent to the cysteinyl β‐carbon of ACV and can then selectively abstract the pro‐*S* hydrogen from this position to form a thioaldehyde/Fe‐peroxide intermediate **24**. This step is irreversible and, in the wildtype enzyme, commits ACV to the oxidative bicyclisation pathway.^47^ The thioaldehyde/Fe‐peroxide can then mediate β‐lactam closure via **25** 20, 21, 48 to generate a monocyclic/ferryl (i.e., Fe^IV^‐oxo) intermediate **26**. Direct evidence for a ferryl species has been reported for other members of the non‐heme iron oxygenase family (i.e., enzymes closely related to IPNS),^49^, ^50^, ^51^ and in model complexes that mimic the active site of non‐heme iron(II) enzymes.^52^ The iron(IV)‐oxo species in **26** can then effect thiazolidine closure through hydrogen atom abstraction from the valinyl β‐carbon to generate radical intermediate **27**, which drives thiazolidine closure to afford the IPN complex **28**. With I325*, we propose that the initial steps of the reaction mechanism proceed as normal, with ACV and O_2_ binding and abstraction of the cysteinyl pro‐*S* β‐H leading to a thioaldehyde/Fe‐peroxide intermediate 24* (Figure 7 b). This species may continue the standard reaction cycle to form IPN **2**, significant quantities of which are generated even with I325*. However, an alternative pathway is available in the absence of the C‐terminal residues, which, as noted previously, would otherwise adopt a position that extends the final helix (α‐10) relative to the Mn:IPNS structure and enclose the substrate in the active site.^6^ Without these residues shielding the reaction centre, the thioaldehyde in 24* is susceptible to attack by a thiolate (or thiol) nucleophile (ACV2) from outside the active site, leading to an enzyme‐bound hemi‐thioacetal/Fe^IV^‐oxo intermediate **29**. In support of the proposed mechanism, one of us has previously reported a high‐yielding reaction in which a thiolate nucleophile attacks a metal‐bound thioaldehyde (formed by hydride abstraction in a model rhenium complex).^53^ The iron(IV)‐oxo species **29** incorporates two ACV moieties covalently linked by a C−S bond. Reaction of the high valent iron(IV)‐oxo intermediate to remove the second cysteinyl β‐hydrogen from the active‐site‐bound ACV1 would afford the iron‐bound thio‐ester species **30**. (This step is very similar to the mechanism believed to operate in the reaction of IPNS with the depsipeptide substrate analogue δ‐(l‐α‐aminoadipoyl)‐l‐cysteine d‐α‐hydroxyisovaleryl ester (AC*O*V), which leads ultimately to a thiocarboxylic acid product.)^15^ In intermediate **30**, there is no longer a thiolate tether between substrate and iron. It seems probable that the absence of this link, combined with increased motility induced by the pendant ACV2 moiety sitting outside the active site, would facilitate departure of the whole “bis‐ACV” product **8** from the active site. Tautomerisation of the dithioester **8** in the reaction buffer would give the observed product **7**.

It seems likely that the thioaldehyde in intermediate 24* would be susceptible to reaction with other extraneous nucleophiles in addition to the thiol(ate) of a second ACV molecule. However any such products have not been observed in significant quantities nor successfully isolated during the current study. Thus, it is possible that the observed ACV2 binding mode reflects, at least in part, a mechanism of capture of ACV by IPNS.

## Conclusions

Substitution of key residues around the substrate binding pocket of IPNS demonstrate that the hydrophobic nature of this binding pocket is vital to the successful progression of IPNS catalysis to form bicyclic products, and its steric profile is highly optimised to the catalytically productive conformation of the native substrate ACV **1**. Even minor steric changes to these residues, such as Leu to Ile or Val, are poorly tolerated, whereas reducing hydrophobicity by introducing Thr in place of Val or Leu is highly deleterious. The geometric constraints imparted by Pro283 are also critical to the catalytic efficiency of the IPNS active site.

Removing the seven C‐terminal residues to create the tailless I325* mutant provides important insight into the mechanism of IPNS catalysis, particularly the role played by these residues in shielding the reactive species generated at the enzyme active site during ACV turnover. It has been observed previously that, in the complex of wildtype IPNS with ACV, these seven residues “adopt a conformation that extends the final helix (α‐10) relative to the Mn:IPNS structure and encloses the substrate in the active site.”^6^ Thus, the C‐terminal residues shield the active site and protect the reactive species present in intermediates **23**–**27** (Figure 7 a) from alternative reaction paths. It follows that, in a mutant enzyme lacking these residues, the active site is more exposed and open. The formation of **7**/**8** from reaction of the tailless I325* mutant with ACV provides direct evidence that reactive intermediates generated in this more accessible active site can be intercepted by alternative reaction partners and diverted from the reaction course seen with the wildtype enzyme. However, the intermolecular reaction with a second ACV that leads to **7**/**8** is presumably disfavoured relative to intramolecular reaction to close the β‐lactam ring (leading in time to IPN), which is why **7** is only formed as a minor by‐product alongside significant quantities of the penam **2**.

It is possible that binding interactions that bring ACV2 near to the active site reflect a mechanism by which IPNS captures ACV and delivers it to the active site. This may in part help to explain the high efficiency of IPNS in the cellular context where it is involved in the large scale commercial production of penicillins. Furthermore, IPNS has a mode of reactivity that is to date unique, and it is likely that this oxidase evolved from the much more prevalent 2‐oxoglutarate (2‐OG) dependent oxygenases. Comparison of IPNS and 2‐OG oxygenase structures reveals that ACV occupies the region of the active site normally involved in 2OG binding. Thus, it is possible that the ACV2 binding site in part reflects the position originally occupied by ACV (or a related substrate) in a 2OG dependent progenitor of IPNS.

In this regard it is notable that ene‐thiols (as present in **7**) are potent metallo‐β‐lactamase (MBL) fold enzyme inhibitors (see Figure 5 c and discussion above).^43^ MBL‐fold enzymes catalyse a range of reactions in addition to β‐lactamase hydrolysis, including nucleic acid hydrolysis and cell detoxification pathways.^54^ Interestingly, the MBL‐fold enzyme ETHE1 (ethylmalonic encephalopathy protein 1) is an iron(III)‐dependent oxygenase that catalyses oxidation of glutathione persulfide to give sulfite,^55^, ^56^, ^57^ a reaction that is closely related to that of I325* IPNS with ACV (though initiating reaction at the H_2_S‐derived sulfur, rather than the cysteinyl β‐carbon as in I325* IPNS).

Finally, the unusual reaction observed with I325* mutant IPNS provides compelling evidence for the proposed involvement of a thioaldehyde intermediate in the reaction cycle of wildtype IPNS. It has long been thought that such a species is involved in this reaction; however, directly characterising it has proved difficult.^15^, ^16^, ^17^, ^18^, ^24^ The dithioester product **7**/**8** reported here is most plausibly formed through interception of a thioaldehyde at the IPNS active site by a second ACV thiol/thiolate (Figure 6 b). Observation of this shunt product is therefore strong support, albeit indirect, for the involvement of a thioaldehyde intermediate in IPNS catalysis.

Conversion of the cysteine‐containing tripeptide **1** to a dithioester product **7**/**8** constitutes a new mode of reactivity for IPNS and non‐heme iron(II) oxidases/enzymes in general. This reaction requires two electron oxidation of the cysteinyl β‐carbon twice, from the CH_2_ carbon of a thiol to the thiocarboxylic acid oxidation state, combined with formation of a second C−S bond at this same carbon centre. IPNS has previously been shown to mediate thiocarboxylate formation at this position in two different substrate analogues,^15^, ^58^ but direct enzymatic formation of a dithioester is unprecedented for this enzyme and others in its class.

## Experimental Section

Chemicals were from Sigma–Aldrich (Poole, UK) or Fluka, except for tris(carboxyethyl)phosphine (Pierce, USA). Chromatography resins were from Pharmacia (now GE Healthcare, UK).

### Production of mutant enzymes

Substitutions were introduced into the wildtype *Aspergillus nidulans* IPNS gene by the unique site elimination method (GE Healthcare, UK), and were confirmed by DNA sequencing. Primers for these mutations are given in Table S1 (Supporting Information). Wildtype *A. nidulans* IPNS was produced as described previously.^5^ In brief, about 50 mg of >90 % pure protein (by SDS‐PAGE), was produced by anion exchange chromatography. For crystallisation, further purification was performed by gel filtration on Sepharose S75 and ion exchange chromatography over MonoQ resin.^5^ This yielded protein of >95 % purity by SDS‐PAGE analysis. Electrospray ionisation mass spectrometry was used to confirm the presence of the predicted mass changes (Table S2, Supporting Information).

ACV **1** was isolated and purified from the crude extract of a fermentation broth of *Cephalosporin acremonium* strain Takeda N2, which is mutated in IPNS.^59^, ^60^, ^61^ This strain is discussed in more detail in the Supporting Information. Incubation of the crude material with a two molar excess of tris(carboxyethyl)phosphine at room temperature^62^ was followed by HPLC on a Hypersil 250×10 mm C_18_ column using a gradient of methanol in aqueous ammonium bicarbonate (10 mm) and UV detector. Fractions containing ACV **1** were freeze‐dried to give a white solid, which was stored at −80 °C until required.

### Enzyme assays and isolation of products

Incubations with IPNS were typically conducted in a final volume of 1 mL, which contained ascorbic acid (1 mm), tris(carboxyethyl)phosphine (TCEP, 1 mm), catalase (0.3 mg mL^−1^), iron(II) sulfate (10 μm), ACV **1** (40 μm), and IPNS (0.2 μm). Incubations were carried out at 28 °C with stirring and aliquots removed at specified time points, mixed with an equal volume of methanol and flash‐frozen in liquid nitrogen before HPLC analysis. Analytical HPLC was carried out on a 250×4.6 mm C_18_ column using a gradient of acetonitrile in aqueous potassium phosphate buffer (25 mm, pH 6.8) at a flow rate of 1 mL min^−1^.^30^ Product quantities were determined by integration of peak areas in the chromatogram of the elution as measured through an absorbance detector at 214 nm. Identification of ACV **1** and IPN **2** was by comparison with authentic standards.

Scaled‐up incubations with the mutant contained ascorbic acid (1 mm), TCEP (1 mm), catalase (0.3 mg mL^−1^), ACV **1** (5 mm), iron(II) sulfate (100 μm), IPNS I325* mutant (10 μm), and ammonium bicarbonate (25 mm) in a final volume of 1 mL. After incubation at 28 °C for 40 min, second aliquots of iron(II) sulfate and TCEP equal to the amounts of each already present were added and incubation was continued for a further 40 min. Products were isolated by subjecting the whole assay mixture to HPLC separation, using a gradient of methanol in 25 mm aqueous ammonium bicarbonate on a 250×10 mm C_18_ Hypersil column. Fractions containing IPN **2** and the new product **7** were collected and freeze dried. The resultant white solids were stored at −80 °C prior to further analysis. If further purification was required, a second HPLC separation was performed using the same gradient of methanol in 50 mm ammonium bicarbonate.

### LC‐MS and NMR analyses

LC‐MS analyses used a gradient of CH_3_CN (2–95 %) in formic acid (0.1 %) on a Jupiter C4 150×4.6 mm column (Phenomenex, UK) at a flow rate of 1 mL min^−1^, eluting via a flow splitter into a Waters ZMD quadrupole electrospray ionisation mass spectrometer in positive ion mode.

For NMR analyses, the new product **7** purified from multiple incubations was pooled. NMR spectra were acquired using a Bruker AVANCE DRX500 spectrometer equipped with a 5 mm ^1^H{^13^C, BB} TBI probe for ^1^H and ^1^H‐^13^C 2D data or on a Bruker AMX500 spectrometer equipped with a 5 mm BBO broadband observe probe for 1D ^13^C data. Samples were prepared in 3 mm tubes in D_2_O (140 μL) with ^1^H and 2D spectra collected at 283 K so that no ^1^H resonances were masked by the solvent peak. 2D gradient, selected COSY, HMQC and HMBC spectra were collected with 2 K *t*_2_ data points over 256 *t*_1_ increments using 8, 128 and 512 transients per increment, respectively (total experiment times of 1, 12 and 52 h, respectively). The 1D ^13^C spectrum was collected for 16 000 transients over a 14 h period.

### Crystallography

Crystals of the IPNS mutant I325* were grown anaerobically, transferred into cryoprotectant solution and flash‐frozen as previously described.^63^, ^64^ Data were collected at 100 K using synchrotron radiation of wavelength 0.93487 Å at beamline ID14‐3 of the European Synchrotron Radiation Facility (ESRF), Grenoble, France equipped with a 165 mm MAR Research CCD detector.

Data were indexed and integrated with MOSFLM^65^ and scaled using SCALA from the CCP4 suite of programs.^66^ An initial structure was generated by rigid body refinement of protein atoms from the IPNS:Fe^II^:ACV model (PDB 1BK0)^6^ against the new data. Further refinement was carried out using REFMAC^67^ and Coot for model building.^68^ The active site iron atom, substrate and water molecules were added in the course of refinement. Crystallographic coordinates and structure factors for the I325* IPNS mutant have been deposited in the Protein Data Bank (PDB) under the accession number 2BJS.

## Conflict of interest

The authors declare no conflict of interest.

## Supporting information

As a service to our authors and readers, this journal provides supporting information supplied by the authors. Such materials are peer reviewed and may be re‐organized for online delivery, but are not copy‐edited or typeset. Technical support issues arising from supporting information (other than missing files) should be addressed to the authors.

Supplementary
