# Supplementary material for: Terminally Truncated Isopenicillin N Synthase Generates a Dithioester Product: Evidence for a Thioaldehyde Intermediate during Catalysis and a New Mode of Reaction for Non‐Heme Iron Oxidases
Source: Chemistry. 2017 Aug 21;23(52):12815–24. doi: 10.1002/chem.201701592 (PMC5637899; doi:10.1002/chem.201701592)
Supplement: Supplementary file 1 — Supplementary [file CHEM-23-12815-s001.pdf]

CHEMISTRY
A European Journal

Supporting Information

Terminally Truncated Isopenicillin N Synthase Generates a Dithioester Product: Evidence for a Thioaldehyde Intermediate during Catalysis and a New Mode of Reaction for Non-Heme Iron Oxidases

Luke A. McNeill,[a, d] Toby J. N. Brown,[a, e] Malkit Sami,[a, f] Ian J. Clifton,[a] Nicolai I. Burzlaff,[b] Timothy D. W. Claridge,[a] Robert M. Adlington,[a] Jack E. Baldwin,[a] Peter J. Rutledge,*[c] and Christopher J. Schofield*[a]

chem_201701592_sm_miscellaneous_information.pdf

Contents

1. Comparison of the Structures of IPNS:Mn(II) and IPNS:Fe(II):ACV Complexes S2
2. Production, Purification and Assays of IPNS Variants S3
3. Additional Data and Characterisation of 'New Product' 7 of the I325* IPNS Variant S6
    a. HPLC Elution Profile from Incubation of I325* IPNS with ACV **1** S6
    b. UV/Vis Spectra of **7** S6
    c. LCMS Data from Reactions of **7** with Dithiothreitol and/ or Iodoacetamide S7
    d. NMR Data for Compound **7** S9
4. Proposed Products of Reactions of I325* IPNS with ACV + Other Peptides S13
5. Crystallographic Data and Statistics S15
6. References S16

1. Comparison of the Structures of Mn:IPNS and IPNS:Fe(II):ACV Complexes

a.

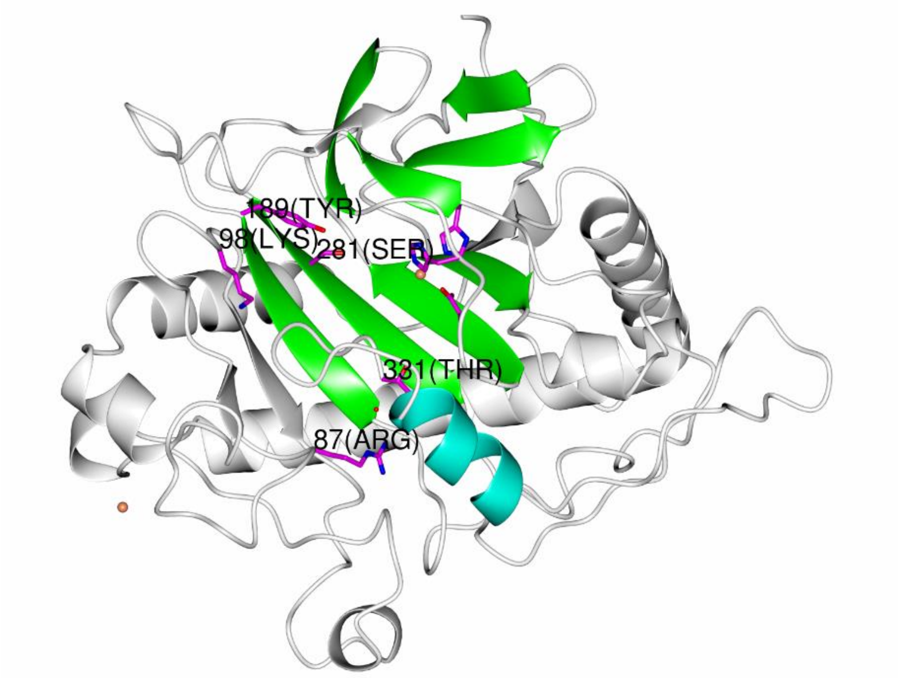

b.

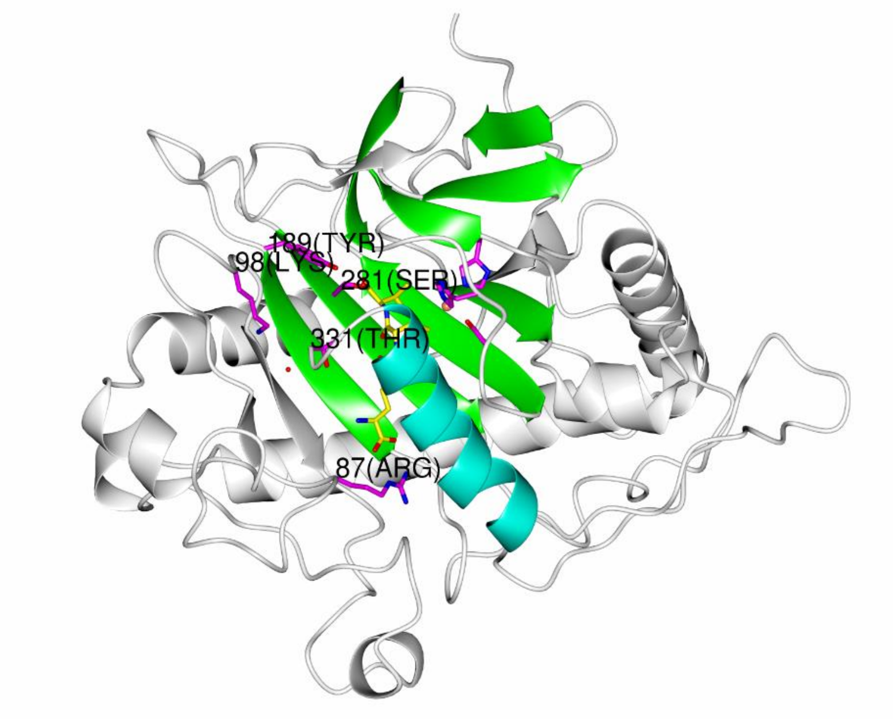

Figure S1: Views of overall structures of **a.** IPNS.Mn (PDB ID 1ips)[S1] and **b.** IPNS:Fe(II):ACV (PDB ID 1bk0),[S2] shown from the same orientation to highlight the core double-stranded β-helix fold (green), and the *C*-terminal α-helical region, residues 313–331 (cyan). Mn(II) is used as a Fe(II) surrogate to enable crystallisation under aerobic conditions. Note the extra turn on the cyan-coloured helix in the second structure, the seven *C*-terminal residues (Asn326–Thr331) having moved upon substrate binding to take up a new conformation that encloses ACV in the active site. The active-site metal ion (iron, or the substitute manganese) is in orange, key substrate-binding residues (Arg87, Tyr189, His214, Asp216, His270, Arg279 and Ser281) are magenta, and ACV **1** is yellow.

2. Design, Production, Purification and Assay of IPNS Variants

Leu223 and Leu231 were substituted to alter steric demand around the substrate valine residue (L223I; L223V; L231I; L231V). Leu231 and Val272 were also altered to introduce an additional hydrogen bonding partner that might enable tighter substrate binding by ligation to the valinyl carboxylate of ACV (L231T; V272T). Pro283 was substituted (P283A; P283V; P283L; P283I) to relax the conformational restrictions imposed by proline and potentially enable formation of cepham products via C–H abstraction from Cγ of ACV valine (in addition to the usual penam product formed from reaction of the tertiary C–H at Cβ). Note the P283L mutation corresponds to the P285L loss-of-function mutation found in IPNS from the *Takeda N2* strain of *C. acremonium* which accumulates ACV and is used for production of the tripeptide in fermentation broths.[S3-5] Lys98, which facilitates rearrangement of the C-terminus upon substrate binding by forming a salt-bridge to Thr331,[S2, 6] was substituted for glutamate to investigate the importance of this Lys98–Thr331 interaction to ACV turnover. To render the change K98E, a triple mutation was introduced, Gly97-Lys98-Lys99 to Arg-Glu-Thr, as suggested by the sequence of IPNS from *S.cattleya* (UniProtKB/Swiss-Prot Accession number Q53932).[S7]

Two larger changes were also introduced. To probe the importance of *C*-terminus mobility, an elongated variant *332Q, ten residues longer at the *C*-terminus than the wildtype enzyme, was made by removing the natural stop codon thus enabling read-through to the in-frame stop codon 30 bases later in the wildtype gene. Finally, the I325* variant , from which the last six residues of the wildtype protein were deleted, was made to investigate the influence of the *C*-terminus on distribution of turnover product .[S8]

Table S1: Primers used in the USE mutagenesis reactions.

Primer	Sequence
Selection: *Hind*III - *Mlu*I	5'-GCC AAA ACA GCC ACG CGT GGC TGC AGC C-3'
L223V	5'-GGA CTG GTA AAC CAC AGT GAT TAG-3'
L223I	5'-GGA CTG GTA GAT CAC AGT GAT TAG-3'
L231V	5'-GC AGT TTC TAC CTG AAC GTT CTG CAC GTT GG-3'
L231I	5'-GC AGT TTC TAC CTG GAT GTT CTG CAC GTT GG-3'
L231T	5'-GC AGT TTC TAC CTG AGT GTT CTG CAC GTT GG-3'
V272T	5'-GC ATT AAC CCA TTT AGT CCG ATG-3'
P283I	5'-GG TTG ACG AAG AAG ATC AGG GAC TGG CGC-3'
P283L	5'-GG TTG ACG AAG AAC ATC AGG GAC TGG CGC-3'
P283V	5'-GG TTG ACG AAG AAT ATC AGG GAC TGG CGC-3'
P283A	5'-GG TTG ACG AAG AAA ATC AGG GAC TGG CGC-3'
K98E†	5'-GAA GGA CTC GAC TGT CTC GCG CCC GGG GAT GGA CAG-3'
*332Q	5'-CCC TCG CTT CTG GGT CTG GCC-3'

† See note in text above regarding the triple-mutation required to render the 'K98E' variant.

The targeted variants were produced and purified to *ca.* 95% purity (determined by SDS-PAGE)and the activity analysed by monitoring IPN **2** formation by HPLC (**Figure 1, main text**).

Table S2: Electrospray ionisation mass spectrometry analyses confirm predicted mass changes wrought by mutation.

Mutant	*Measured mass (Da)*	*Predicted mass (Da)*	*Difference (Da)*
Wildtype	37393	37391	2
L223V	37379	37377	2
L223I	37391	37391	0
L231V	37379	37377	2
L231I	37393	37391	2
L231T	37387	37379	8
V272T	37396	37393	3
P283I	37404	37407	-3
P283L	37410	37407	3
P283V	37400	37393	7
P283A	37372	37371	1
K98E	37348	37449	-101[†]
*332Q	38261	38260	1

† An $[M–101]^+$ ion was observed at proportions varying from 10% to 65% of the ion corresponding to the predicted mass in the spectra of all variants, and as a major peak in the spectrum of K98E protein. The abundance of the $[M–101]^+$ ion could be altered by varying the conditions used for protein preparation and MS analysis (data not shown). This ion is thought to arise from cleavage of the C-terminal residue (Thr331). To test this hypothesis, a sample of a different protein (P283V variant) was treated with carboxypeptidase Y; this led to the same change ([M–101]) in the mass spectrum, supporting the proposal that this ion arises via loss of Thr331 from the *C*-terminus. This hydrolysis is apparently promoted by the changes in structure wrought by the K98E triple mutation.

The Effects of Active Site Substitution on IPNS Activity

Changing either Leu223 or Leu231 to Ile or Val had similar effects on activity, reducing it to ~30–35% of that observed for wildtype IPNS. Reducing the steric bulk of the *substrate* in a similar way, i.e. by substituting the valine residue of the tripeptide substrate for alanine, eliminates the ability of IPNS to form a β-lactam product: substrate analogue L-δ-(α-aminoadipoyl)-L-cysteinyl-D-alanine (ACA) is not turned over to a β-lactam product by IPNS, even though it could in theory form a bicyclic penam product via thiazolidine closure onto the methyl side-chain of D-Ala.[S9-10] Crystal structures for the IPNS:Fe(II):ACA complex indicate the presence of additional water molecules in the active site that are not present in the ACV complex, and it is proposed that these either interfere with binding of the cosubstrate dioxygen, or divert key intermediates in the reaction cycle preventing ACA turnover.[S9] It is possible that a similar effect is in operation with these sterically reduced IPNS mutants, in that hydrophobic interactions between the enzyme and substrate are

altered in a way that significantly hampers turnover, or additional water molecules are able to access the binding pocket and interfere with catalysis.

Introducing Thr in place of Val at position 272 had a similar level of effect on activity, reducing it to ~ 30% of that of the wildtype. However, replacing Leu231 with Thr had a more deleterious effect, almost completely eliminating IPN formation. Both Leu231 and Val272 are highly conserved across IPNS enzymes from different species,[S4] however the reasons behind the different outcome of these two mutations are not obvious. In the IPNS:Fe(II):ACV structure (1BK0), the two protein side-chains are a comparable distance from the valinyl isopropyl group (*ca.* 4.0 Å at distance of nearest approach) and from iron (*ca.* 6.5 Å), although the side-chain of Val272 is closer to the ACV valine carboxylate (*ca.* 4.3 Å versus *ca.* 5.4 Å), the putative hydrogen bonding partner for the Thr mutants. Whatever the reason, this outcome indicates that the hydrophobicity of different regions of this binding pocket are differentially important to catalysis.

Pro283 substitution reduced activity more dramatically than substitution of Leu223, Leu231 or Val272. The P283A/V/I variants had *ca.* 10–12% of the wildtype activity, and the P283L showed no activity, even in the holed-plate bioassay for antibiotic activity (which can detect activity at levels below those discernible by HPLC).[S11-12] As noted above, P283L corresponds to the mutation found in the *Takeda N2* mutant strain of *C. acremonium*, which knocks out IPNS activity.[S3] The lack of IPNS activity by the *Takeda N2* mutant allows high levels of ACV to accumulate, and the tripeptide is easily isolated as a mixture of thiol and disulphide species. Notably, P283L also showed a marked propensity to precipitate from solution, suggesting that this mutation triggers a change in protein conformation which likely also contributes to the lack of activity.

The K98E mutant was designed to eliminate the capacity of this side-chain to form a salt-bridge with the C-terminal carboxylate of Thr331 when ACV binds to the enzyme, and this mutation lowers catalytic activity to *ca.* 10% of the wildtype enzyme. This result provides further support for the proposal that movement of the C-terminal tail is important in IPNS catalysis.

Further, the activity of the *332Q mutant is severely reduced (~5% of wildtype), demonstrating that adding residues to the IPNS *C*-terminus – and likely impeding its movement – can be damaging to catalysis. We have previously shown that successive deletion of amino acids from the C-terminus of IPNS results in progressive loss of activity,[S8] and the *332Q result provides a further indication of the importance of this region.

3. Additional Data and Characterisation of 'New Product' 7 of I325* IPNS variant

a. HPLC Elution Profile from Incubation of I325 IPNS with ACV **1***

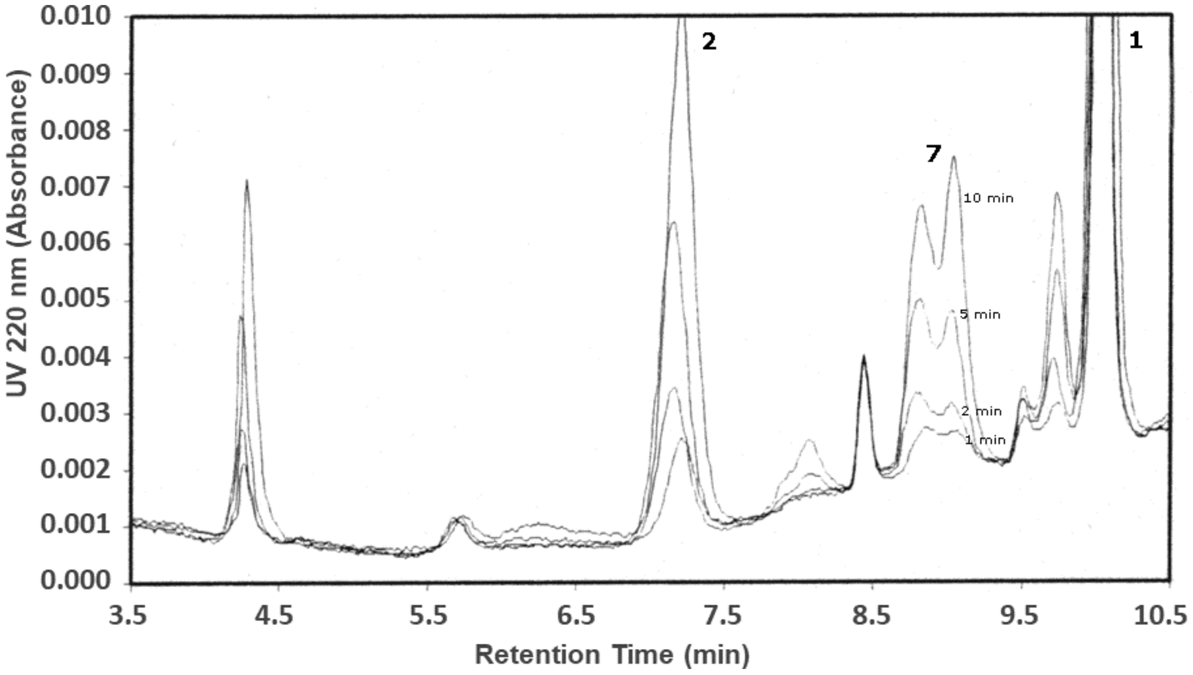

Figure S2: Elution profiles (C_{18} column, see Experimental section for HPLC conditions) of the reaction mixture from turnover of ACV **1** by I325* IPNS after 1, 2, 5 and 10 min incubation at 28 °C. IPN **2** (R_t = 7 min) was identified by its antibiotic activity, ACV **1** (R_t = 10 min) by isolation and ^{1}H NMR spectroscopy. $[ACV]_2$ (disulphide) is also present and elutes at R_t = 14 min. The peak for new product **7** (R_t ~ 9 min) increases with time as indicated. The split peak was not resolvable and was collected as a single fraction. Subsequent analysis showed this mixed fraction to contain only a single compound, consistent with equilibrating thio-keto/enol tautomers observed in NMR experiments.

*b. UV/Vis Spectra of **7***

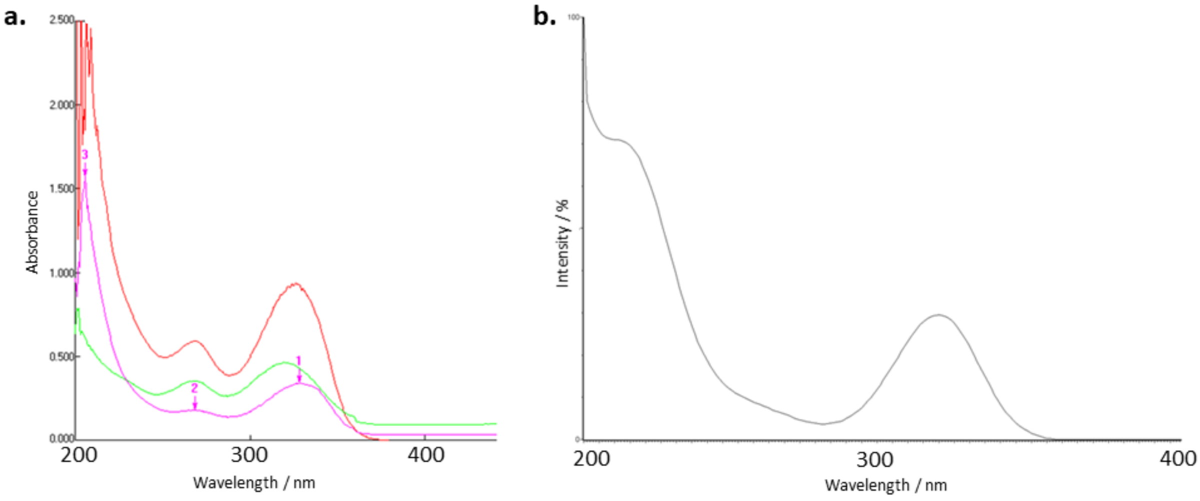

Figure S3: UV spectra of the new product **7**. **a**. Spectra acquired during HPLC purification (solutions of **7** in ammonium bicarbonate buffer, see Experimental); the red, green and magenta traces are from three separate HPLC fractions; the numbers 1, 2 and 3 on the figure indicate the three main absorbance bands, at λ_{max} = 317, 270 nm and 210 nm respectively. **b.** Absorbance spectrum for the peak shown in Figure S3a (i.e. compound **7**), showing

absorbance at λ_{max} = 317 nm. (The LCMS runs shown in Figure S4 were also monitored with a diode array detector, from which the data shown in Figure S3b were extracted.)

*c. LCMS Data from Reactions of **7** with Dithiothreitol and/ or Iodoacetamide*

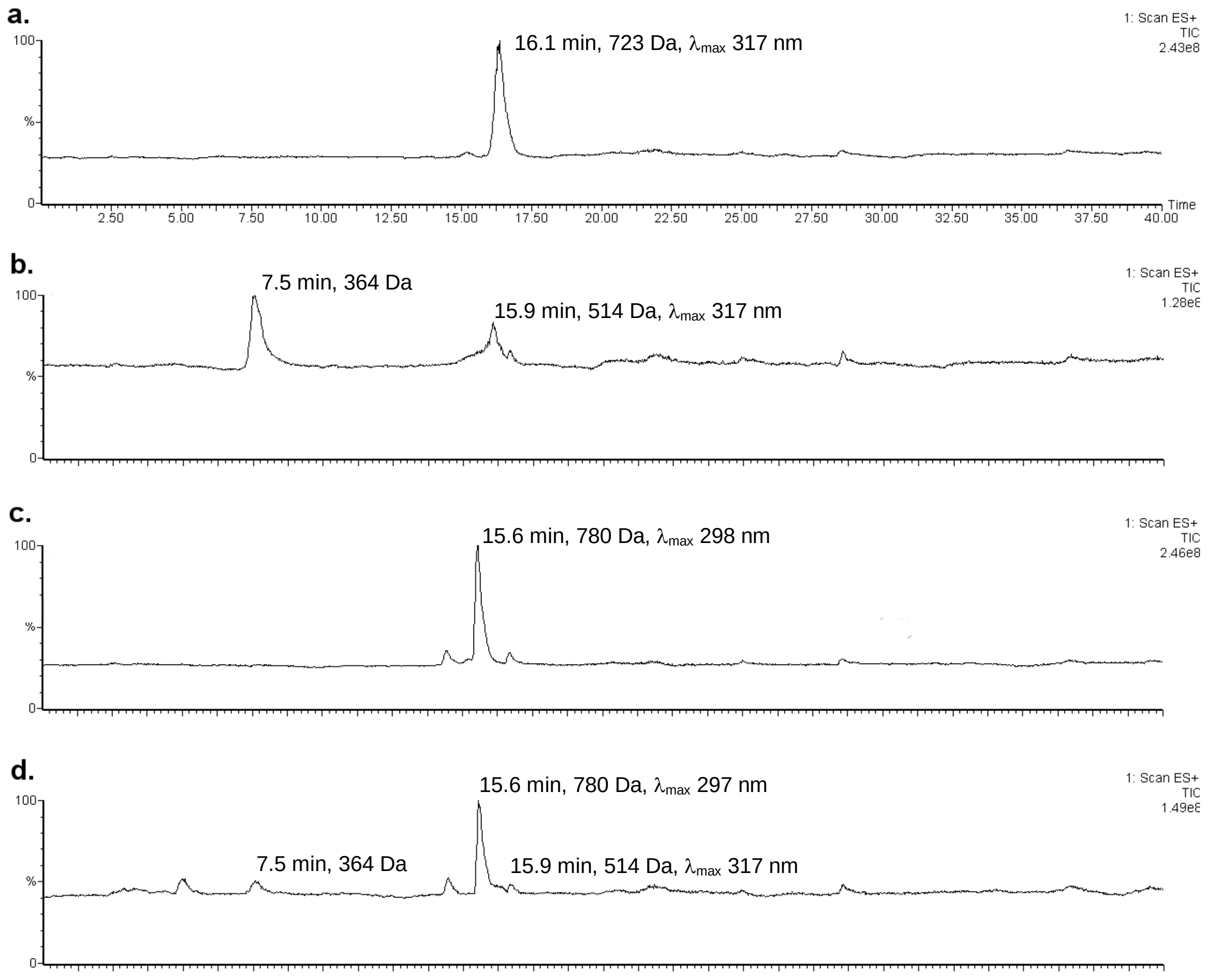

Figure S4: Total ion count (TIC) traces of the elution from a C4 HPLC column of the reaction of **7** with DTT and iodoacetamide. Masses are quoted as $[MH]^+$ ion. In each reaction, a 50-fold excess of iodoacetamide, DTT or both were added to the new compound **7** and the mixture incubated at room temperature for 30 minutes prior to analysis. **a. 7** only, **b. 7** + DTT, **c. 7** + iodoacetamide, **d. 7** + DTT + iodoacetamide. Proposed structures of the species formed are shown in **Figure S5**. Assignment of peaks is shown in **Table S3**.

Table S3: Assignment of key peaks in the mass spectra shown in **Figure S3**.

Retention time (min)	Mass $[MH]^+$ (Da)	λ_{max} (nm)	Assignment	Appears in trace
7.5	364		ACV **1**	b. and d.
15.6	780	297	**7**:IAc Adduct	c. and d.
15.9	514	317	**7**:DTT Adduct	b. and d.
16.1	723	317	**7**	a

Only major peaks are assigned. Minor peaks in the traces c. and d. presumably represent isomers of the major products or hydrolysis products.

a.

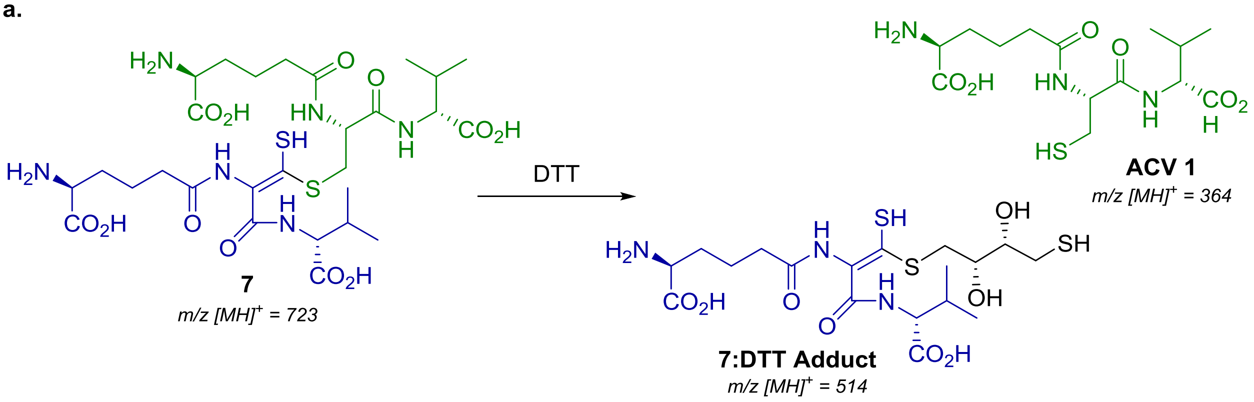

b.

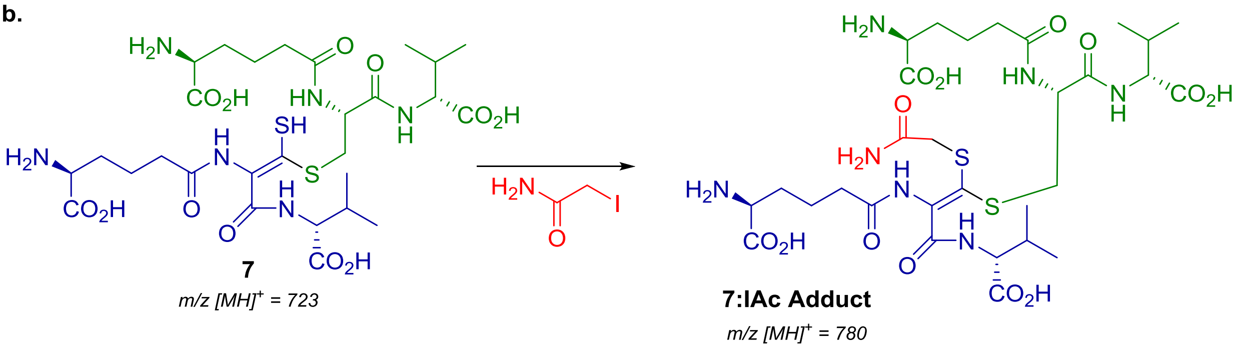

Figure S5: Proposed products formed from reactions of **7** with **a.** dithiothreitol (DTT) and **b.** iodoacetamide (IAc). (Note that some bond lengths are exaggerated to improve the clarity of the overall structure, and that the alkene stereochemistry in **7** and adducts is not defined – see Figure 3 and legend in main text.) The 'simple' disulfide adduct of ACV **1** and DTT (RS–SR′, *m/z* $[MH]^+$ = 516) was not observed under the conditions used for this experiment. Note that the *E/Z*-stereochemistry of the alkene in **7** and adducts **7:DTT** and **7:IAc** is not defined (see Figure 3, legend and discussion in the main manuscript), and the stereochemistry at all other positions is assumed to be as in ACV **1**.

*d. NMR Data for Compound **7***

Table S4: Assignment of chemical shifts in the ^{1}H and ^{13}C NMR spectra of **7**. Chemical shifts were recorded from ^{1}H, HMQC and HMBC spectra at 283 K (for full spectra see Figures S6–S8) and are referenced to residual CH_3OH resonances at 3.340 ppm (^{1}H) and 49.5 ppm (^{13}C). Shifts quoted for doublets, triplets, multiplets etc represent the centre of the splitting pattern. As noted in the main text (see Figure 3, legend, and discussion of ACV 'double oxidation'), **7** is an oxidised dimer of two molecules of ACV **1**. One ACV moiety (green in Figure 3 and below) is *intact* but for its thiol S–H and is denoted *ACV (I)* in the structure and table below; the other ACV (blue in Figure 3 and below) has been *modified* (by way of double-oxidation at its cysteine) so is denoted *ACV (M)*.

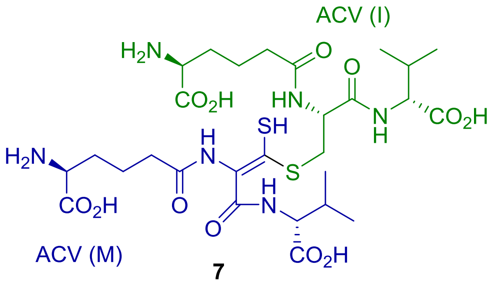

7

**Intact ACV (I)**		**^{13}C (ppm)**	**^{1}H (ppm)**	**Modified ACV (M)**		**^{13}C (ppm)**	**^{1}H (ppm)**
**A$^{(I)}$**	COOH	175.1	–	**A$^{(M)}$**	COOH	175.1	–
	α	55.1	3.739		α	55.1	3.739
	β	30.5	1.875		β	30.5	1.875
	γ	21.6	1.704		γ	21.6	1.704
	δ	35.5	2.349		δ	35.2	2.486
	ε	176.6	–		ε	176.2	–
**C$^{(I)}$**	CO	171.4	–	**C$^{(M)}$**	CO	166.8	–
	α	51.9	4.780		α	–†	–†
	β	37.5	3.867, 3.619		β	229.7	–
**V$^{(I)}$**	COOH	178.6	–	**V$^{(M)}$**	COOH	178.6	–
	α	61.4	4.093		α	61.8	4.041
	β	31.4	2.126		β	31.4	2.126
	γ_1‡	19.7	0.869		γ_1‡	19.6	0.908
	γ_2‡	17.9	0.871		γ_2‡	17.9	0.877

† Not observed due to H/D exchange; see text for further discussion.
‡ Methyl assignments γ_1/ γ_2 are arbitrary.

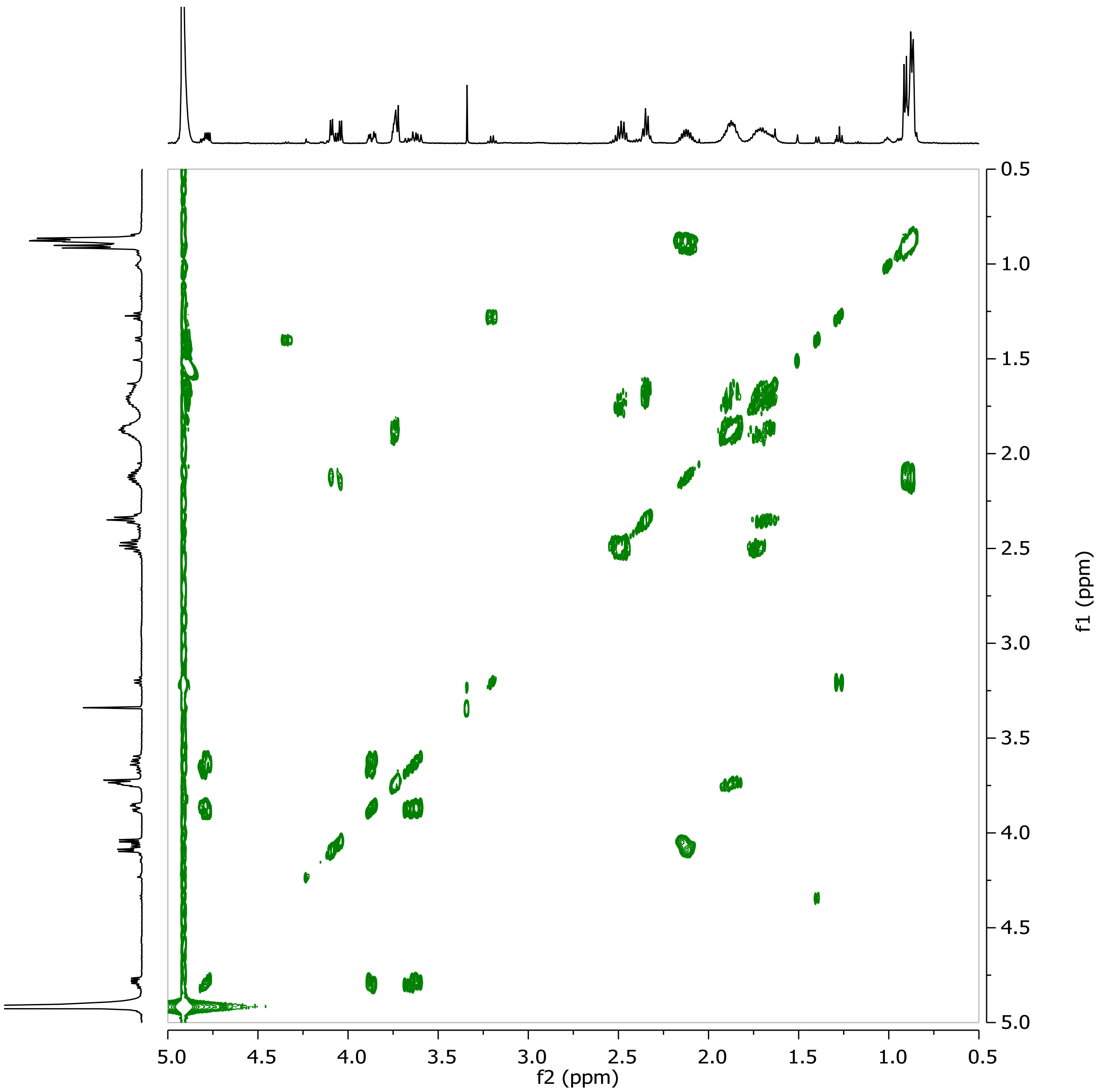

Figure S6: ^{1}H COSY spectrum of **7**

a.

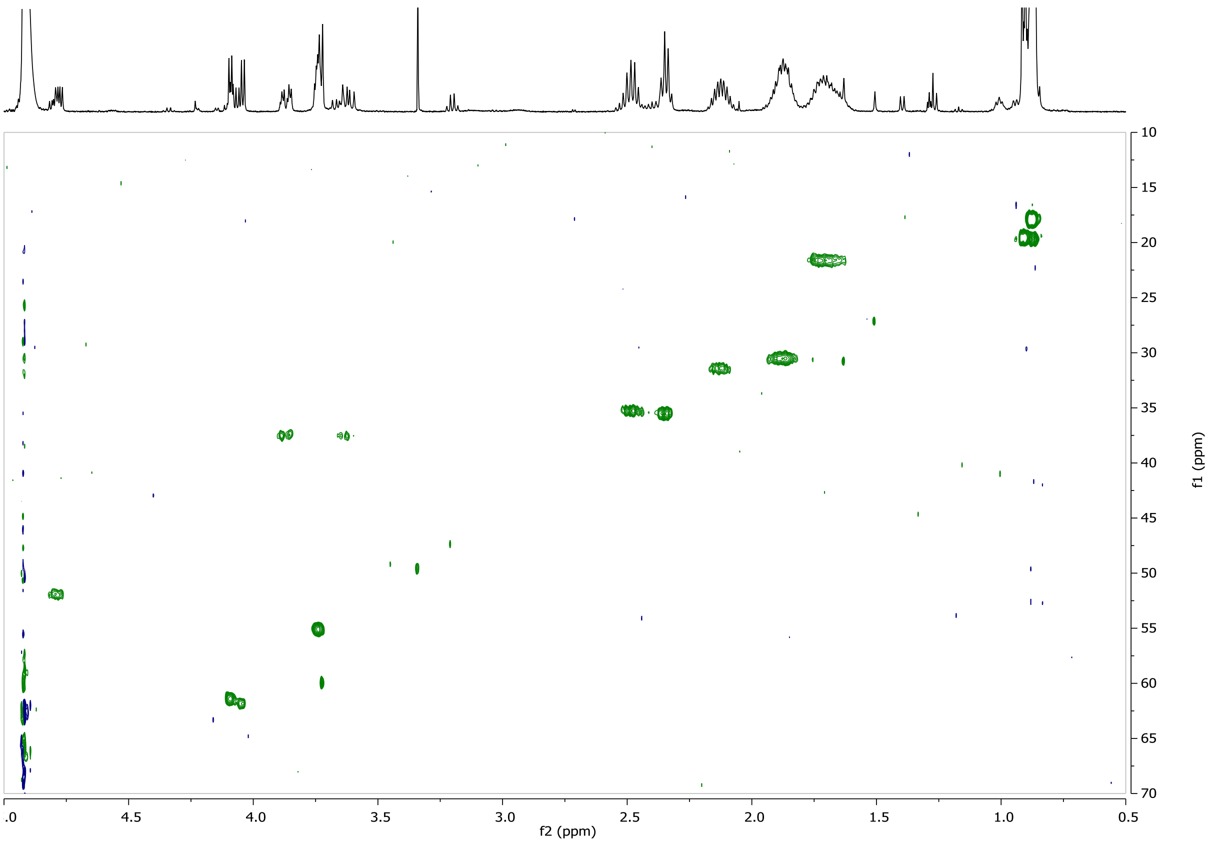

b.

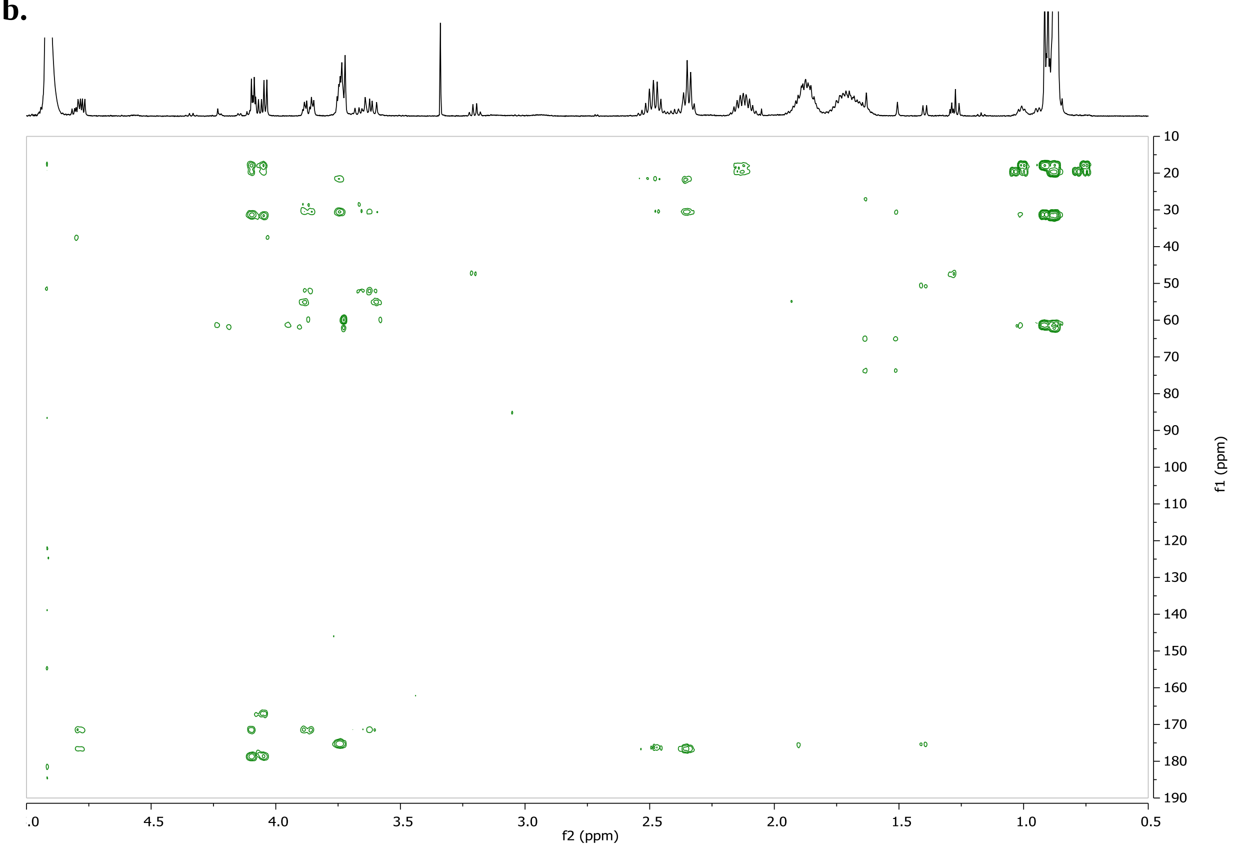

Figure S7: a. ^{1}H-^{13}C HMQC and **b.** ^{1}H-^{13}C HMBC spectra of **7**.

a.

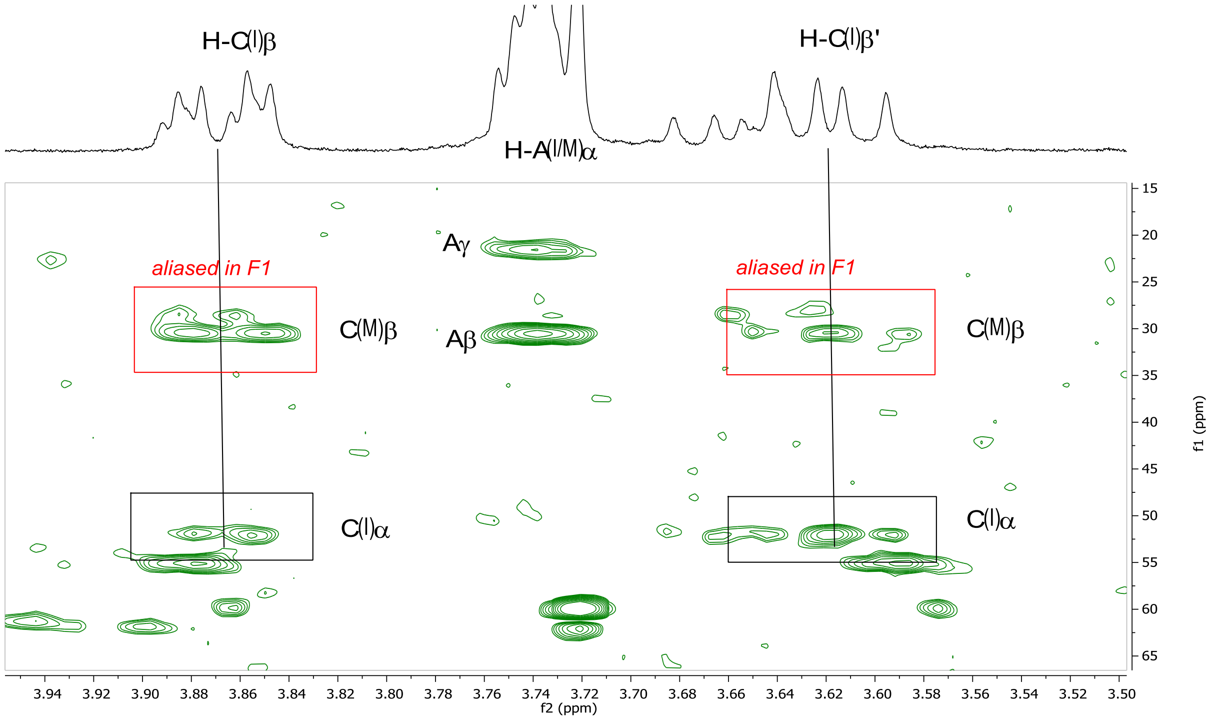

b.

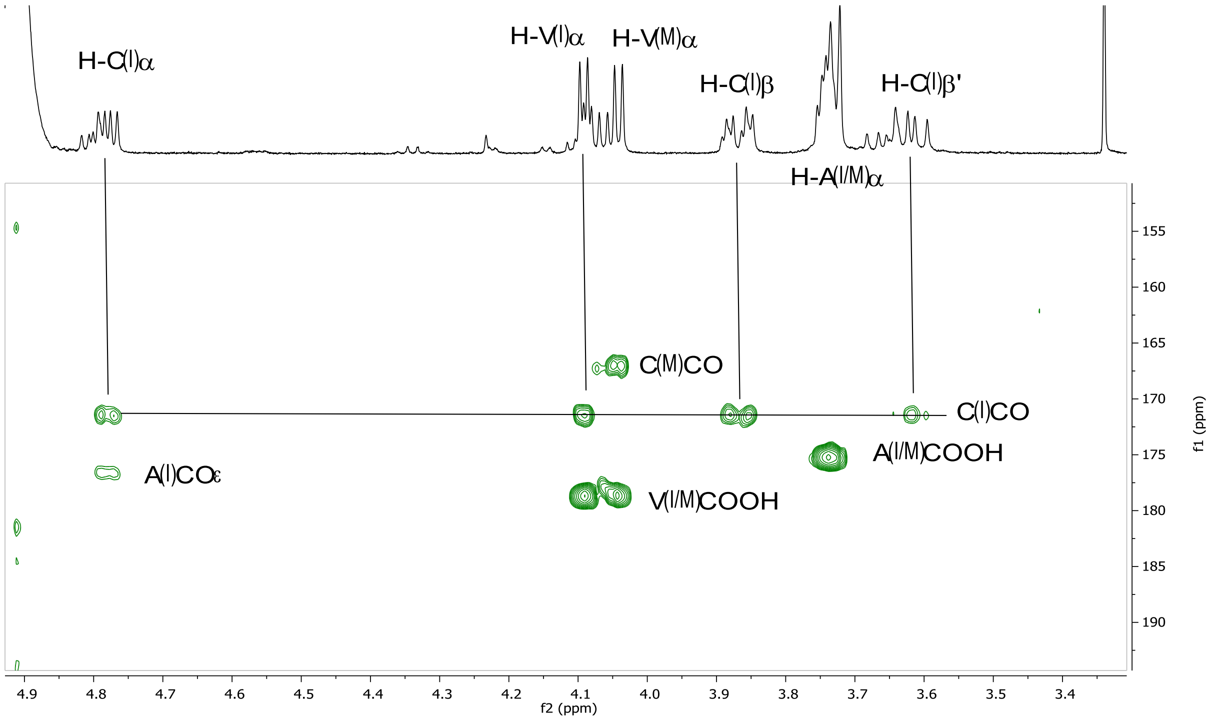

Figure S8: Expansions of the HMBC spectrum showing **a.** correlations of C$^{(I)}$β protons, and **b.** selected correlations to carbonyl groups. The correlations to C$^{(M)}$β highlighted in red are aliased in the ^{13}C (F1) dimension due to the high shift of the sulfur-bearing carbon and thus appear at the incorrect ^{13}C shift. Correcting for this aliasing yields a ^{13}C chemical shift of 229.7 ppm for the C$^{(M)}$β carbon. This shift is in accord with that expected for a dithioester functionality (this carbon is predicted to resonate at 227 ppm by Mnova software, www.mestrelab.com). The annotations (I) and (M) denote the two ACV subunits as in Table S4 above.

4. Proposed Products of Reactions of I325* IPNS with ACV + Other Peptides

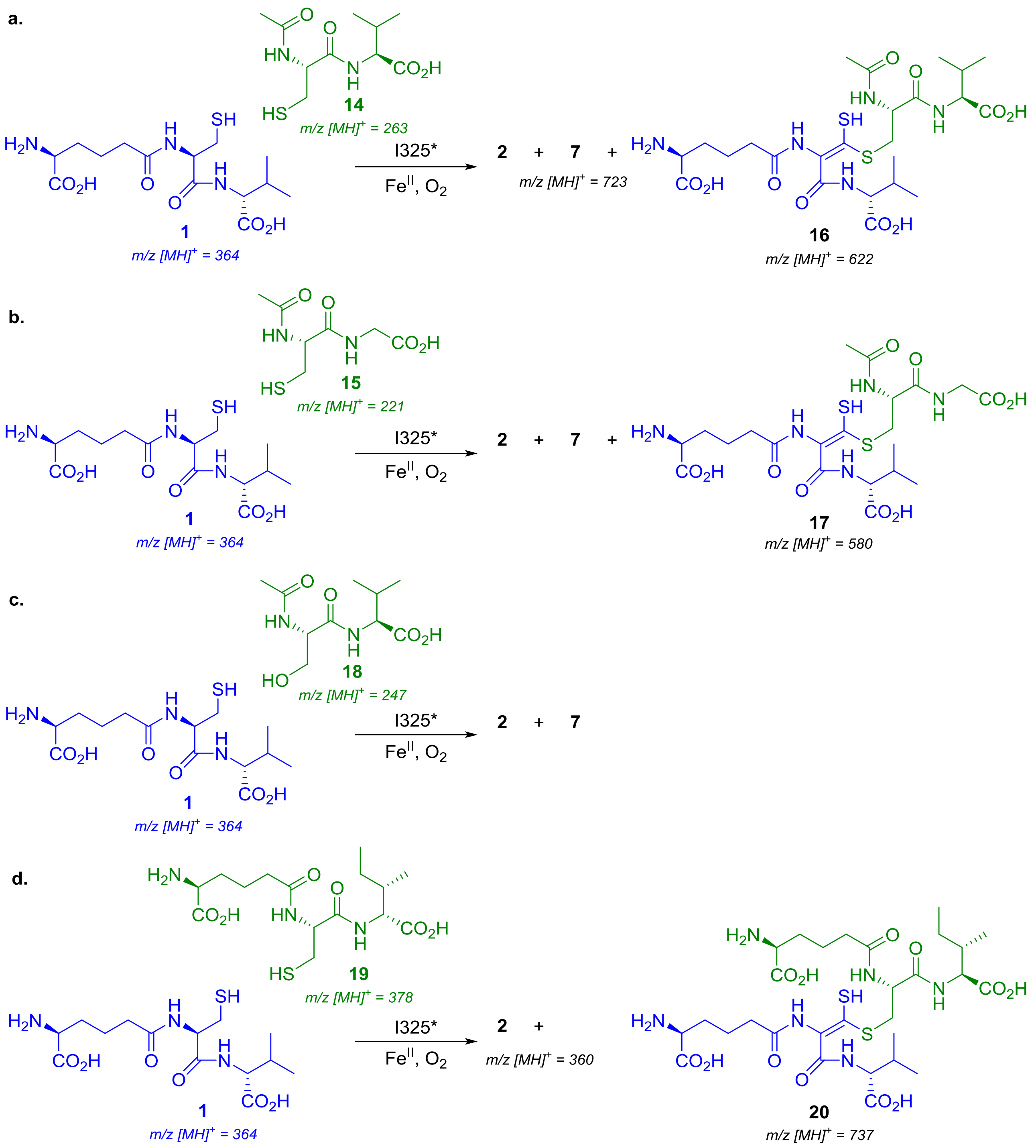

Figure S9: Further reactions of the tailless I325* variant. **a.**, **b.** Reactions with both ACV **1** and the simplified substrate analogues *N*-acetyl-L-Cys-L-Val **14** and *N*-acetyl-L-Cys-Gly **15** afford IPN **2**, the ACV derived thio-ene-thiol 'dimer' **7**, and analogous products **16** and **17** in which oxidised ACV is combined with each of the dipeptide thiols. **c.** The analogous serine derivative **18** does not contain a thiol and does not form the corresponding thio-ene-thiol product. **d.** Reaction of ACV **1** and tripeptide substrate analogue L-δ-(α-aminoadipoyl)-L-cysteinyl-*D*-*allo*-isoleucine **19** gives rise to the alternative ene-thiol **20**. Note that the alkene stereochemistry in **16**, **17** and **20** is not defined (see Figure 3 and legend in main text), and that 'simple' disulfide adducts (RS–SR′) of ACV **1** with **14**, **15** and **18**, and of **19** with itself, were not observed under the conditions used for this experiment. Key LCMS data for these experiments are summarized in Table S5 below.

Table S5: Retention time (R_t) and *m/z* data for major peaks in the LCMS traces arising from reactions of the tailless I325* variant with both ACV **1** and another peptide: **a.** *N*-acetyl-L-Cys-L-Val **14**; **b.** *N*-acetyl-L-Cys-Gly **15**; **c.** *N*-acetyl-L-Ser-L-Val **18**; and **d.** L-δ-(α-aminoadipoyl)-L-cysteinyl-*D*-*allo*isoleucine **19**. See Figure S9 for the structures of compounds **14**–**20**.

	**R_t (min)**	***m/z* [MH]$^+$**	**Assignment**
**a.**	3.8	360	**2**
	7.7	364	**1**
	12.8	263	**14**
	16.5	723	**7**
	17.5	622	**16**
**b.**	3.3	221	**15**
	3.9	360	**2**
	14.5	580	**17**
	16.5	723	**7**
**c.**	3.9	360	**2**
	16.5	723	**7**
**d.**	3.9	360	**2**
	7.6	364	**1**
	14.9	378	**19**
	17.5	737	**20**

5. Crystallographic Data and Statistics

Table S6: Data collection and statistics for the I325* variant IPNS, (PDB ID 2BJS).

X-ray source	ESRF ID14-3	
Wavelength λ (Å)	0.93487Å	
Space group	$P2_12_12_1$	
Unit cell (*a* , *b*, *c* Å)	47.149 71.126 100.991	
Resolution shell (Å)#	30.00 – 1.30	1.37 – 1.30
Measurements	310035	39865
Average I/σI	14.6	4.3
Unique reflections	82674	11815
Completeness (%)	98.4	97.7
R_{merge} (%)§	5.9	31.3
R_{cryst} (%)*	14.16	
R_{free} (%)†	16.51	
RMS deviation¶	0.026 Å (2.2°)	
B factors‡ (Å^2)	16.9, 19.6, 6.4, 10.6, 30.8, 9.4, 25.3	
Redundancy≠	3.80 (3.40)	
Residues	320	
Water molecules	546	
Ramachandran outliers∀	0	
Ramachandran favoured	97.2%	
Poor rotamers	0	
Favoured rotamers	96.7%	
MolProbity score	1.25	

# Statistics shown for high-resolution pass. A low-resolution pass was merged with the data.

§ $R_{merge} = \sum_j \sum_h | I_{h,j} - \langle I_h \rangle | / \sum_j \sum_h \langle I_h \rangle \times 100$

* $R_{cryst} = \sum | |F_{obs}| - |F_{calc}| | / \sum |F_{obs}| \times 100$

† R_{free} = based on 4% of the total reflections.

¶ RMS deviation from ideality for bonds (followed by the value for angles).

‡ Average *B* factors in order: main chain, side chain, substrate ACV1, ACV2, solvent, iron, sulfate.

≠ Redundancy (followed by redundancy in outer shell)

∀ Quality indicators calculated using Molprobity.[S13]

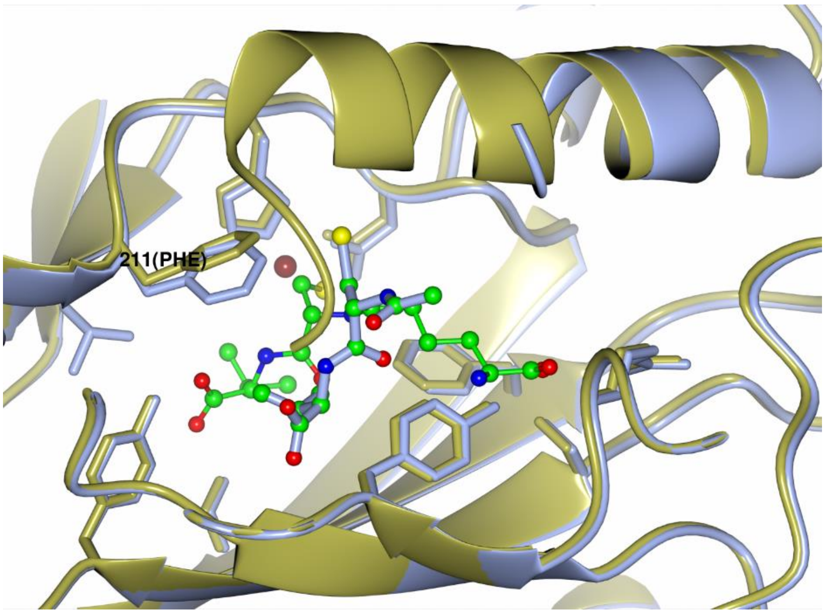

Figure S10: Overlay figure to highlight the change in position of Phe211 in I325* IPNS (2bjs, protein in gold with substrate in green), relative to the wildtype IPNS:Fe(II):ACV structure (1bk0, in lilac). The sidechain of Phe211 undergoes a rotation in the mutant structure relative to the wildtype. It has previously been proposed that this sidechain helps to shield the active site – see main text for further details. The interested reader is referred to further discussion of this point in the context of IPNS-mediated turnover of the unsaturated substrate analogue L-δ-(α-aminoadipoyl)-L-cysteinyl-*D*-vinylglycine, specifically Figure 3 and discussion thereof in this earlier paper.[S14]

6. References

[S1] P. L. Roach, I. J. Clifton, V. Fulop, K. Harlos, G. J. Barton, J. Hajdu, I. Andersson, C. J. Schofield, J. E. Baldwin, *Nature* **1995**, *375*, 700-704.

[S2] P. L. Roach, I. J. Clifton, C. M. H. Hensgens, N. Shibata, C. J. Schofield, J. Hadju, J. E. Baldwin, *Nature* **1997**, *387*, 827-830.

[S3] M. Ramsden, B. A. McQuade, K. Saunders, M. K. Turner, S. Harford, *Gene* **1989**, *85*, 267-273.

[S4] G. Cohen, D. Shiffman, M. Mevarech, Y. Aharonowitz, *Trends Biotechnol.* **1990**, *8*, 105-111.

[S5] J. F. Martín, S. Gutiérrez, *A. van Leeuw. J. Microb.* **1995**, *67*, 181-200.

[S6] P. L. Roach, I. J. Clifton, C. M. H. Hensgens, N. Shibata, C. J. Schofield, J. Hajdu, J. E. Baldwin, *RCSB Protein Data Bank* **1998**, *1BK0*.

[S7] Y. Wang, R. Li, *Wei Sheng Wu Xue Bao* **1996**, *36*, 87-92.

[S8] M. Sami, T. J. N. Brown, P. L. Roach, C. J. Schofield, J. E. Baldwin, *FEBS Lett.* **1997**, *405*, 191-194.

[S9] A. J. Long, I. J. Clifton, P. L. Roach, J. E. Baldwin, P. J. Rutledge, C. J. Schofield, *Biochemistry* **2005**, *44*, 6619-6628.

[S10] P. Graf, PhD thesis, Eidgenössischen Technischen Hochschule (Zürich), **1986**.

[S11] C. P. Pang, B. Chakravarti, R. M. Adlington, H. H. Ting, R. L. White, G. S. Jayatilake, J. E. Baldwin, E. P. Abraham, *Biochem. J.* **1984**, *222*, 789-795.

[S12] J. E. Baldwin, J. M. Blackburn, J. D. Sutherland, M. C. Wright, *Tetrahedron* **1991**, *47*, 5991-6002.

[S13] V. B. Chen, W. B. Arendall, III, J. J. Headd, D. A. Keedy, R. M. Immormino, G. J. Kapral, L. W. Murray, J. S. Richardson, D. C. Richardson, *Acta Crystallogr. Sect. D* **2010**, *66*, 12-21.

[S14] J. M. Elkins, P. J. Rutledge, N. I. Burzlaff, I. J. Clifton, R. M. Adlington, P. L. Roach, J. E. Baldwin, *Org. Biomol.Chem.* **2003**, *1*, 1455-1460.
